# PD-L1^+^ neutrophils at the invasive margin drive contact-dependent CD8^+^ T cell exhaustion in hepatic alveolar echinococcosis

**DOI:** 10.3389/fimmu.2026.1917606

**Published:** 2026-09-10

**Authors:** Dalin Shi, Chengwei Tie, Mingquan Pang, Xiaolei Xu, Zhixin Wang, Haijiu Wang, Haining Fan

**Affiliations:** 1 Qinghai Research Key Laboratory for Echinococcosis, Qinghai University, Xining, Qinghai, China; 2 Central Laboratory, Affiliated Hospital of Qinghai University, Xining, Qinghai, China; 3 Qinghai University School of Clinical Medicine, Qinghai University, Xining, Qinghai, China; 4 Department of Hepatopancreatobiliary Surgery, Affiliated Hospital of Qinghai University, Xining, Qinghai, China

**Keywords:** CD8^+^ T cell exhaustion, hepatic alveolar echinococcosis, PD-L1/PD-1 axis, PD-L1^+^ neutrophils, spatial immune niche

## Abstract

**Introduction:**

Hepatic alveolar echinococcosis (HAE), a lethal chronic helminth infection caused by Echinococcus multilocularis, is characterized by pronounced local immune evasion and progressive CD8+ T cell exhaustion at the parasite?host invasive margin. However, the spatial cellular network and core regulatory subsets driving this localizedimmunosuppression remainpoorly understood.

**Methods:**

We performed high-resolution spatial immune profiling of the lesion invasive margin (collagenous layer tissue, CLT) and paired distal normal liver tissues (DLT) from 12 HAE patients by integrating imaging mass cytometry, single-cell RNA sequencing, and multiplex immunofluorescence. The underlying molecular mechanism was validated via in vitro co-culture assays with or without Transwell physical separation, and the therapeutic potential was further confirmed in a delayed?treatment HAE mouse model using neutrophil depletion(anti-Ly6G) or PD-L1 blockade (anti-PD-L1).

**Results:**

Spatial multi-omics identified a specific subset of APC-like PD-L1 neutrophils exclusively enriched at the HAE invasive margin, which showed striking spatial co-localization and frequent membrane-to-membrane contacts with PD-1 CD8 T cells. In vitro, activated CD8 Tcells potently induced PD-L1 upregulation on neutrophils; reciprocally, PD-L1 neutrophils significantly suppressed T cell effector function (downregulating GZMB, IFN-γ, and TNF-α) and upregulated exhaustion-associated transcription factors TOX and NR4A1 via a contact-dependent PD-L1/PD-1 pathway, effects largely abolished by Transwell separation. In the HAE mouse model, both neutrophil depletion and PD-L1 blockade significantly reduced parasite burden, alleviated liver inflammation and fibrosis, and restored CD8 T cell cytotoxicity; notably, neutrophil depletion achieved superior therapeutic efficacy compared with PD-L1 monotherapy.

**Conclusion:**

Collectively, our findings demonstrate that PD-L1-expressing neutrophils at the HAE invasivefront contribute importantly to localized CD8 T cell exhaustion via direct cell-cell contact, anddisrupting this crosstalk represents a promising targeted strategy to restore host protective immunity against parasitic infection.

## 1 Introduction

Larval infection of *Echinococcus multiloculari*s leads to hepatic alveolar echinococcosis (HAE). This lethal neglected tropical disease spreads invasively within liver tissues, similar to malignant tumors (1). The prevalence of HAE exceeds 1.6% in high-risk areas of the Qinghai-Tibet Plateau, bringing heavy pressure to regional medical services (2, 3). *Albendazole* is the main drug for HAE treatment at present. However, liver toxicity and gradually emerging drug resistance restrict its long-term application, so new immunotherapies are urgently required (4, 5).

In the setting of chronic parasitic infection, sustained antigenic stimulation drives CD8^+^ T cells toward a state of functional exhaustion, marked by upregulated inhibitory receptors (PD-1, TIM-3, TIGIT) and impaired secretion of effector cytokines. *TOX* serves as the central transcriptional regulator of this process, with upstream axes such as *CXCR4/JAK2/STAT3* gradually uncovered in recent years. In HAE, however, the cellular triggers that initiate this exhaustion program at the lesion-host interface remain undefined (6, 7). Recent studies have further revealed that CXCR4 can regulate the TOX mediated CD8+ T cell exhaustion program through the JAK2/STAT3 signaling pathway, and blocking this axis can effectively prevent functional T cells from acquiring a depleted phenotype (8). In experimental chronic infection models of *Trypanosoma cruzi*, PD-1^+^ TOX^+^ CD8^+^ T cells emerge specifically during the chronic disease phase, and the severity of T cell exhaustion correlates positively with infection duration (9). Accumulating evidence has established the PD-1/PD-L1 axis as a central driver of CD8^+^ T cell exhaustion across a broad spectrum of chronic infection settings, including viral infections (HIV, HBV, LCMV) and parasitic diseases such as *leishmaniasis* (10).

Consistent with these findings, the PD-1/PD-L1 axis has also been implicated in mediating CD8^+^ T cell dysfunction in both clinical HAE specimens and *E. multilocularis*-infected mouse models; blockade of this pathway partially rescues T cell effector function and attenuates parasite burden *in vivo* (11). Targeting additional co-inhibitory receptors such as TIGIT has likewise been shown to reverse T cell exhaustion and suppress lesion progression in preclinical HAE studies (12). Despite these advances, the specific cell subsets that initiate T cell exhaustion via direct spatial crosstalk at the lesion invasive margin—the primary interface of intense host-parasite interaction in HAE—have yet to be fully characterized.

Neutrophils, as an important component of the hepatic immune microenvironment, have attracted much attention in recent years for their immunomodulatory function in chronic inflammation (13, 14). Beyond their canonical bactericidal role, neutrophils can polarize into an immunosuppressive phenotype with high PD-L1 expression under chronic inflammatory conditions. This contact-dependent regulatory mode has been extensively described in tumor microenvironments, yet whether a similar mechanism operates at the parasite-host interface in HAE has not been directly investigated. Given the prominent neutrophil infiltration at the HAE invasive margin, we speculate that PD-L1^+^ neutrophils may act as an underrecognized driver of localized T cell dysfunction (15, 16).

**Figure 1:**
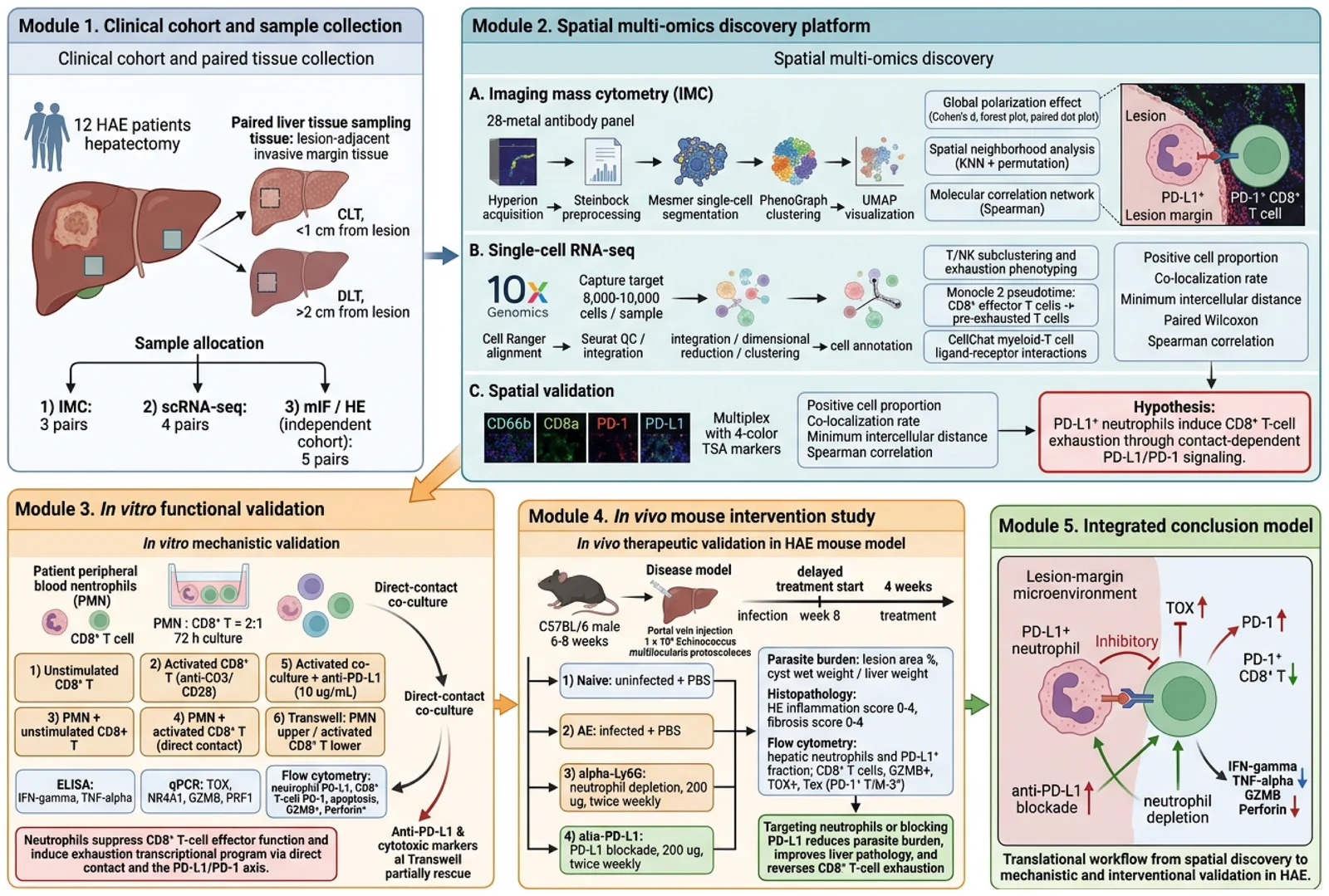
Overall study design and experimental workflow. This study integrates spatial multi-omics, in vitro mechanistic validation, and in vivo therapeutic intervention to elucidate the immune escape mechanisms at the invasive margin of hepatic alveolar echinococcosis (HAE). Module 1: Clinical cohort. Paired collagenous layer tissue (CLT) and distal liver tissue (DLT) were collected from 12 patients with HAE undergoing hepatectomy. Module 2: Spatial multi-omics discovery. Imaging mass cytometry (IMC) and single-cell RNA sequencing (scRNA-seq) were utilized to map the spatial immune landscape and identify key cell-cell interactions, including the PD-1/PD-L1 and TGF-β signaling axes. Multiplex immunofluorescence (mIF) was performed for spatial validation of key findings. Module 3: In vitro functional validation. Co-culture assays using primary neutrophils and CD8 T cells were conducted to verify the contact-dependent immunosuppressive mechanism mediated by PD-L1 neutrophils. Module 4: In vivo therapeutic intervention. The therapeutic efficacy of neutrophil depletion (α-Ly6G) and PD-L1 blockade (α-PD-L1) was evaluated in a mouse model of alveolar echinococcosis (AE). Module 5: Integrated model. PD-L1 neutrophils drive CD8 T cell exhaustion at the HAE invasive margin, and targeting this axis represents a promising therapeutic strategy for HAE.

Based on these observations, we hypothesize that PD-L1^+^ neutrophils enriched at the invasive margin of HAE lesions activate the PD-L1/PD-1 axis via direct spatial contact with CD8^+^ T cells, thereby driving *in situ* T cell exhaustion. To test this hypothesis, we integrated imaging mass cytometry (IMC), single-cell RNA sequencing (scRNA-seq) and multiplex immunofluorescence (mIF) to construct a high-dimensional single-cell spatial atlas of the lesion invasive margin. Functional validation was further performed using *in vitro* co-culture assays and HAE mouse models, aiming to elucidate the spatial organizational principles of HAE immune escape and provide novel evidence for the development of precision targeted immunotherapy strategies. The overall study design and experimental workflow are illustrated in **Figure 1**.

## 2 Materials and methods

### 2.1 Clinical samples and ethics

All study protocols were reviewed and approved by the Ethics Committee of Qinghai University Affiliated Hospital on March 8, 2025 (approval No. P-SL-2025-032). Written informed consent was obtained from all enrolled patients and healthy volunteers prior to biological sample collection. Baseline clinicopathological characteristics of the 12 recruited patients with HAE are summarized in **Table 1**.

**Table 1:** Clinical characteristics of HAE patients included in the study.

Characteristic	Value (n=12)
Age, years, mean ± SD	36.8 ± 15.2
Sex, male/female	6/6
Disease duration, months, median (IQR)	36.0 (12.0-84.0)
WHO PNM stage	
II	6 (50.0%)
III	2 (16.7%)
IV	4 (33.3%)
Lesion size, cm, mean ± SD	15.8 ± 3.8
Prior treatment	
Albendazole	5 (41.7%)
None	7 (58.3%)
Liver function	
ALT, U/L, median (IQR)	37.5 (33.0-47.5)
AST, U/L, median (IQR)	32.0 (23.0-39.0)
Albumin, g/L, mean ± SD	34.8 ± 4.5
Sample allocation	
IMC (paired CLT+DLT samples)	3
scRNA-seq (paired CLT+DLT samples)	4
mIF/HE (additional paired validation samples)	5

All 12 patients provided paired invasive marginal tissue (CLT) and distal normal liver tissue (DLT) samples. The PNM staging was based on the World Health Organization (WHO) classification of hepatic alveolar echinococcosis. IMC, Imaging mass spectrometry; scRNA-seq, Single-cell RNA sequencing; mIF, Multiplex immunofluorescence; HE, Hematoxylin-eosin. Continuous variables that conform to normal distribution are expressed as mean ± standard deviation; Variables with non-normally distributed variables are represented by median (interquartile range, IQR). The classification variable is expressed in the number of cases (percentage).

We enrolled 12 patients diagnosed with HAE via imaging and pathological examination. Inclusion criteria: Diagnosed as primary hepatic alveolar echinococcosis by preoperative abdominal imaging (ultrasound, CT/MRI) and confirmed by postoperative histopathology; Aged 8–70 years, regardless of gender; Underwent elective radical hepatectomy in our hospital, with complete paired specimens of lesion invasive margin and distal normal liver tissue available; Patients with preoperative albendazole treatment were eligible, provided that the drug was discontinued for at least 7 days before surgery. Exclusion criteria: Combined with viral hepatitis (HBV, HCV), liver cirrhosis, autoimmune liver disease, hepatocellular carcinoma or other malignant tumors; Combined with other parasitic diseases, systemic acute infection or active inflammatory diseases; Received preoperative immunotherapy, glucocorticoids, immunosuppressants or chemoradiotherapy within 3 months before surgery (routine albendazole anti-parasitic treatment excluded); Severe dysfunction of heart, lung, kidney or other major organs; Incomplete clinical or pathological data.

Tissues were classified based on their spatial proximity to the lesion. The collagenous layer tissue (CLT)—defined as the invasive margin within a 1 cm radius of the lesion boundary—was compared with distal liver tissue (DLT), collected from non-lesional areas >2 cm from the margin. We allocated 3 paired samples to Imaging Mass Cytometry (IMC), 4 to single-cell RNA sequencing (scRNA-seq), and 5 to multiplex immunofluorescence (mIF) validation. The allocation ratio (3:4:5) was based on the technical requirements of each platform (IMC requiring high-quality FFPE sections, scRNA-seq requiring fresh tissue viability, and mIF for independent validation). Detailed individual clinical data and sample usage information of all enrolled patients are listed in **Supplementary Table 1**.

### 2.2 Single-cell RNA sequencing

Single-cell suspensions were prepared by digesting fresh CLT and DLT specimens in buffer containing 0.5 mg/mL type IV collagenase and 0.1 mg/mL DNase I. Trypan blue staining confirmed ≥85% viability; only suspensions meeting this threshold were carried forward. Libraries were built using the 10x Genomics platform, with a target of 8,000–10,000 cells and an average sequencing depth of approximately 50,000 reads per cell. Raw sequencing data were processed according to a standard 10x Genomics workflow with optimized parameters for clinical liver tissue. Cells with mitochondrial read content above 20% were discarded as low-quality events. Dimensionality reduction and unsupervised clustering were performed using Seurat v4.3 with a resolution of 0.5, including data normalization, highly variable gene screening and UMAP embedding. Detailed cell subset annotation information is listed in **Supplementary Table 6**.

### 2.3 Imaging mass cytometry and multiplex immunofluorescence

Metal-labeled antibody staining was performed on formalin-fixed paraffin-embedded (FFPE) sections of 3 HAE patients (panel containing CD66b, CD8a, PD-1, PD-L1, HLA-DR, α-SMA, etc.). The Hyperion system collected image (1 μm resolution, 35 channels). Steinbock (v0.4.0) image processing and Mesmer (DeepCell) single-cell segmentation were applied. Effect sizes of marker expression differences between paired CLT and DLT regions were calculated using Cohen’s d, with statistical significance assessed by paired Wilcoxon tests (no multiple correction due to the exploratory pilot design). For spatial neighborhood analysis, K-nearest neighbor algorithm (K = 15) was applied, and enrichment significance was determined via 1,000 permutation tests as previously established. Spearman rank correlation was used for biomarker correlation. To corroborate the IMC-derived spatial patterns, mIF was performed on FFPE tissue blocks from both patients and established animal models. Multispectral images were acquired to quantify co-localization of PD-1, CD8a, CD66b, and PD-L1, thereby delineating the immune niche characteristic of the HAE invasive front. Detailed information on the metal-conjugated antibodies used for IMC staining is provided in **Supplementary Table 2**.

### 2.4 *In vitro* co-culture assay

To elucidate the functional mechanisms underlying the spatial interaction between polarized neutrophils and CD8^+^ T cells at the HAE lesion margin, we established an *in vitro* co-culture system using primary peripheral blood neutrophils from HAE patients and purified CD8^+^ T cells from healthy donors, with a neutrophil-to-T cell ratio of 2:1. Contact dependence was verified via Transwell physical separation, and the contribution of the PD-L1/PD-1 axis was confirmed using neutralizing antibody intervention.

Neutrophil isolation from HAE patients: Peripheral blood neutrophils were isolated from enrolled HAE patients by density gradient centrifugation using Polymorphprep™ (Axis-Shield, Norway) strictly following the manufacturer’s protocol. Briefly, fresh ethylenediaminetetraacetic acid (EDTA)-anticoagulated peripheral blood was carefully layered onto an equal volume of Polymorphprep solution, and centrifuged at 500 × g for 30 min at room temperature with the brake turned off. The granulocyte layer corresponding to neutrophils was aspirated, washed twice with pre-cooled phosphate-buffered saline (PBS), and then treated with hypotonic lysis buffer to remove residual erythrocytes. Purity was verified by flow cytometry using CD11b and Ly6G surface markers. All batches achieved > 85% neutrophil purity, and cell viability was confirmed to be > 95% by trypan blue exclusion staining.

CD8^+^ T cell isolation from healthy donors: CD8^+^ T cells were purified from peripheral blood mononuclear cells (PBMCs) of age- and sex-matched healthy volunteers (n=6, 3 males and 3 females, aged 25–45 years) by magnetic-activated cell sorting (MACS, Miltenyi Biotec, Germany) according to the standard operating procedure. All enrolled donors had no history of chronic infection, autoimmune diseases, malignant tumors, or recent medication use within 3 months, and routine blood test indicators were all within the normal reference range. The final purity of isolated CD8^+^ T cells was confirmed to be > 95% by flow cytometry.

CD8^+^ T cells from healthy donors with a uniform baseline functional state were selected for this study. Peripheral blood CD8^+^ T cells from HAE patients exhibit heterogeneous pre-exhausted phenotypes with large individual differences, which would introduce substantial confounding factors and make it difficult to accurately quantify the immunosuppressive intensity of neutrophils on initially activated T cells. This design is a widely accepted paradigm in the field of neutrophil immunoregulation research; the corresponding methodological limitations are further discussed in the Discussion section.

T Cell Activation: Prior to co-culture, purified CD8^+^ T cells were activated with CD3/CD28 magnetic beads (Thermo Fisher Scientific, USA) at a bead-to-cell ratio of 1:1, and cultured in complete RPMI 1640 medium supplemented with 10% fetal bovine serum, 100 U/mL penicillin-streptomycin, and 100 U/mL recombinant human interleukin-2 (IL-2) for 24 h.

Experimental groups:(1) Activated CD8^+^ T cells alone;(2) Activated CD8^+^ T cells co-cultured with neutrophils;(3) Activated CD8^+^ T cells co-cultured with neutrophils + 10 μg/mL anti-PD-L1 neutralizing antibody;(4) Transwell co-culture(neutrophils and CD8^+^ T cells physically separated by 0.4 μm pores, allowing only soluble factor exchange).

Pre-experiments confirmed that the PBS vehicle used to dilute anti-PD-L1 antibody exerted no significant effects on IFN-γ and TNF-α secretion by CD8^+^ T cells (*P* > 0.05 vs antibody-free blank control). Given the limited availability of primary peripheral blood immune cells from HAE patients, no independent isotype-matched IgG control was included in formal co-culture assays to retain sufficient cell numbers for biological replicates.

Flow cytometry was applied to determine the protein expression levels of granzyme B (GZMB), interferon-γ (IFN-γ) and PD-1 in CD8^+^ T cells. Cell apoptosis was detected via Annexin V/PI double staining, while the mRNA transcript levels of *TOX* and *NR4A1* were quantified by reverse transcription quantitative real-time PCR (RT-qPCR). All flow cytometric data were processed and analyzed using FlowJo software (version 10.9.0). Detailed primer sequences and optimized reaction conditions for RT-qPCR assays are provided in **Supplementary Table 8**.

### 2.5 Animal model and therapeutic intervention

All animal experimental procedures were reviewed and approved by the Institutional Animal Care and Use Committee (IACUC) of the Affiliated Hospital of Qinghai University (Approval No. PSL-2025-032) and were conducted in accordance with institutional and national guidelines, the ARRIVE guidelines, and the AVMA Guidelines for the Euthanasia of Animals (2020 edition).

We used 6–8 week-old male C57BL/6 mice to establish the chronic HAE model. Only male mice were selected to avoid gender-related immune variability. For surgical inoculation, mice were anesthetized with isoflurane using a precision vaporizer with 100% oxygen as the carrier gas at a flow rate of 1 L/min: 3–4% for induction and 1.5–2% for maintenance. Anesthetic depth was confirmed by loss of the righting reflex and absence of the pedal withdrawal reflex. A total of 1 × 10^4^ Echinococcus multilocularis protoscoleces were injected into the liver parenchyma according to established and widely adopted protocols in the HAE research field (12, 17).

Rationale for infection route and inoculum dose: The intrahepatic protoscolex injection strategy was prioritized over oral egg gavage for standardized therapeutic evaluation. Oral infection results in low and unpredictable modeling efficiency (30–40% success rate), highly heterogeneous lesion number, size and anatomical distribution, and a prolonged pre-patent period, which introduces substantial variability and reduces statistical power for interventional comparisons (17). In contrast, direct intrahepatic injection achieves >95% modeling success rate, highly uniform lesion distribution and synchronized disease progression, which is the standard protocol for preclinical therapeutic evaluation of HAE in most published studies (12, 17). The inoculum dose of 1 × 10^4^ protoscoleces was selected based on published dose-gradient studies and our own pilot validation: this dose reliably induces progressive fibrotic lesions and pronounced CD8^+^ T cell exhaustion by 8 weeks post-infection, without causing acute lethal toxicity, and is consistent with multiple authoritative HAE interventional studies (3, 12).

Rationale for treatment initiation at week 8: Previous longitudinal studies have demonstrated that E. multilocularis lesions undergo an initial slow-growth phase in the first 6 weeks, followed by a rapid progressive phase starting at 6–7 weeks post-infection, accompanied by accumulated fibrosis and established immunosuppressive microenvironment. We therefore initiated therapeutic intervention at week 8, when chronic fibrotic lesions and CD8^+^ T cell exhaustion are fully established. This delayed-treatment design mimics the clinical scenario in which HAE patients are diagnosed at an already chronic stage, and allows evaluation of therapeutic reversal of established disease rather than prophylactic prevention. We performed qualitative macroscopic observation of liver lesions at week 8 before intervention to confirm uniform disease establishment across all infected mice, but did not conduct systematic quantitative detection of parasite burden, pathological score or immune profiles at this pre-treatment time point to avoid additional surgical trauma and animal loss.

We acknowledge that this surgical inoculation route cannot fully recapitulate the natural oral egg ingestion and oncosphere hepatic migration process of human alveolar echinococcosis, which is noted as a limitation in the Discussion section.

Eight weeks after inoculation, mice were randomly assigned to four cohorts (*n* = 6 per group): (1) naive group (healthy mice, intraperitoneal PBS); (2) AE model group (infected mice, isotype-matched IgG control); (3) α-Ly6G group (infected mice, 200 μg anti-Ly6G antibody intraperitoneally, twice weekly); (4) α-PD-L1 group (infected mice, 200 μg anti-PD-L1 antibody intraperitoneally, twice weekly). All treatments lasted for four consecutive weeks.

At the end of the intervention, mice were deeply anesthetized with 5% isoflurane and euthanized by cervical dislocation. Death was verified by permanent cessation of respiration and heartbeat, and absence of response to a tail pinch. Liver tissues were harvested immediately for parasite burden assessment, histopathological examination, flow cytometry and mIF staining.

### 2.6 Statistical analysis

We adopted Shapiro-Wilk and Levene’s tests to examine data distribution and variance homogeneity. All quantitative results are shown as mean ± SD or mean ± SEM based on data characteristics. Paired Wilcoxon signed-rank tests were used to compare paired CLT and DLT tissues, and Mann-Whitney U tests were adopted for comparisons between two independent groups. We applied one-way ANOVA combined with Tukey’s *post-hoc* tests for datasets following normal distribution, and Kruskal-Wallis tests with Dunn’s *post-hoc* tests for non-parametric data.

Spearman correlation analysis was used to explore correlations among immune molecules. Differential genes from scRNA-seq data were screened via Wilcoxon rank-sum tests with Benjamini-Hochberg correction (*FDR* < 0.05). We ran 1000 permutation tests to calculate the significance of spatial enrichment.

All statistical analyses adopted two-sided tests at a significance level of *P* < 0.05. Data processing and visualization were completed with GraphPad Prism 10.6 and Python 3.11. All original numerical data can be found in **Supplementary Data 1**.

## 3 Results

### 3.1 IMC demonstrates spatial enrichment of PD-L1^+^ neutrophils at the HAE invasive margin

#### 3.1.1 Global polarization: neutrophil accumulation and coordinated upregulation of the PD-L1/PD-1 axis

**Figure 2:**
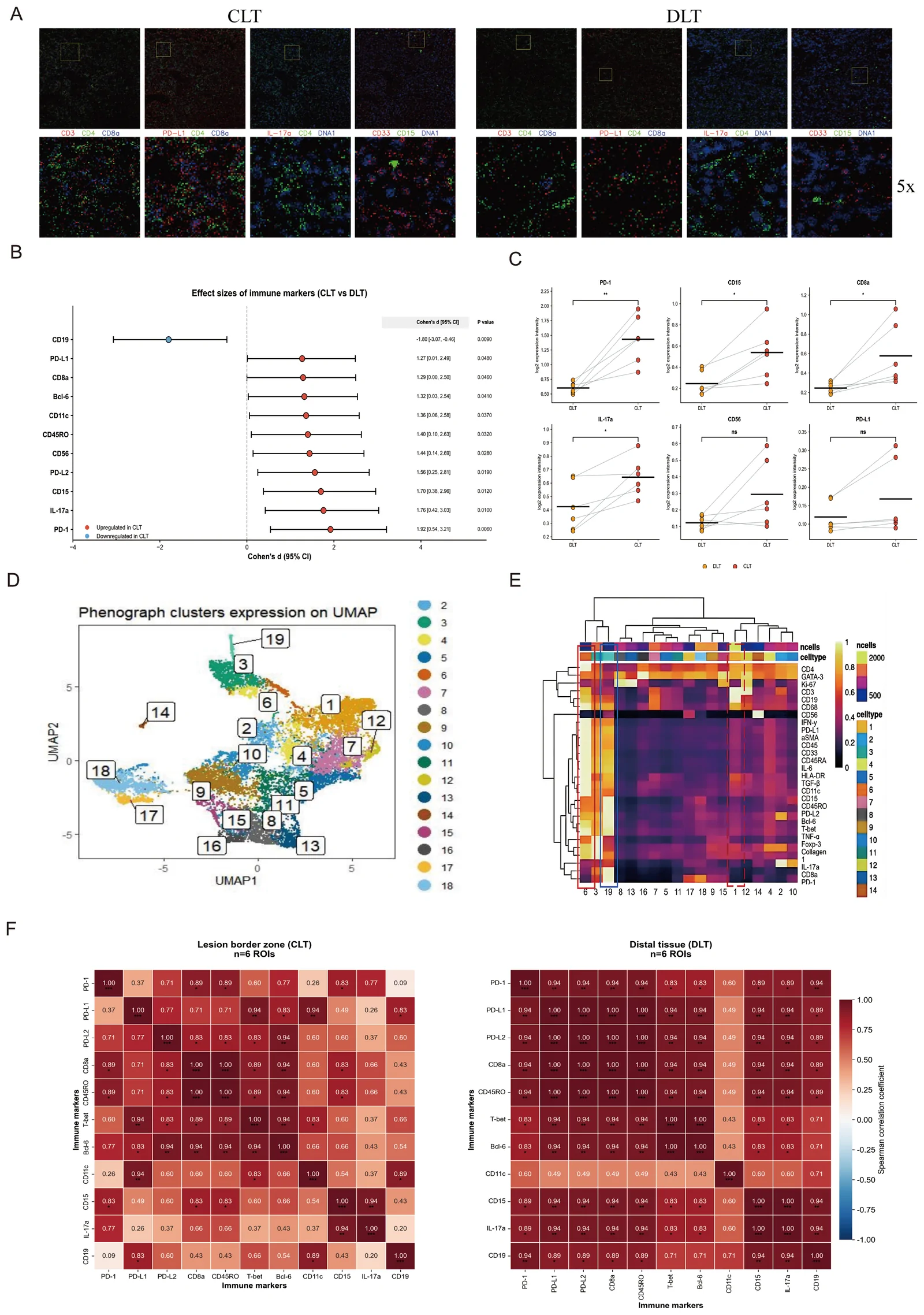
Spatial immune landscape of the invasive margin in hepatic alveolar echinococcosis revealed by imaging mass cytometry. **(A)** Representative IMC pseudocolor images showing the expression and spatial distribution of immune markers in CLT and DLT regions. Upper panels: overview (5× magnification); lower panels: high-magnification view (20× magnification) of the yellow boxed regions. Individual markers are shown in separate channels as indicated below each panel. Nuclei were stained with DNA intercalator (blue). Scale bars: 500 μm (upper panels), 100 μm (lower panels). **(B)** Forest plot representing effect sizes (Cohen’s d) and 95% confidence intervals of immune marker expression differences between paired CLT and DLT regions. Red dots indicate significantly upregulated markers in CLT; blue dots indicate significantly downregulated markers. **(C)** Paired dot plots showing the expression levels of selected immune markers in paired CLT and DLT regions from 6 HAE patients. Data are presented as paired dot plots; each line connects samples from the same patient. Horizontal lines represent median values. *P < 0.05, **P < 0.01, ns: not significant (paired Wilcoxon signed-rank test). **(D)** UMAP visualization of Phenograph clustering of all single cells identified by IMC. A total of 19 distinct cell clusters were identified based on the expression of 35 immune markers. **(E)** Heatmap representing the normalized expression levels (z-score) of 35 immune markers across the 19 Phenograph clusters. The color scale represents the z-score of marker expression. The right vertical bar indicates the number of cells in each cluster. **(F)** Spearman correlation heatmaps demonstrating pairwise correlations between immune markers in CLT (left) and DLT (right) regions. The color scale represents the Spearman correlation coefficient (*r*). Red indicates positive correlation; blue indicates negative correlation. Exact Spearman’s rank correlation coefficients and P values for all marker pairs are provided in **Supplementary Table 3**-**2**.

This study used IMC to analyze the immune microenvironment at the HAE invasion front. Three paired samples of CLT and DLT were collected from patients. The IMC experimental workflow, representative multi-channel staining images and cell area quantification results are shown in **Supplementary Figure 1**. The pseudocolor image (**Figure 2A**) showed a large infiltration of CD15^+^ neutrophils around the fibrotic cyst tissue, often closely adjacent to CD8+ T cells.

To correct for individual differences in patients, we constructed a linear mixed effects model analysis on multiple regions of interest (ROIs) (**Figure 2B**). Compared to remote liver tissue, the expression of programmed death receptor 1 (PD-1) was significantly enriched in pericystic fibrotic tissue (Cohen’s *d* = 1.92; 95% CI: 0.54-3.21; *P* = 0.006) indicating a large effect size. Immune activity markers such as CD15, CD8a, IL-17a are also highly enriched in pericystic fibrotic tissue, with absolute effect sizes *|d|* ≥ 0.8 for each indicator.

Paired t-test confirmed that compared to distal liver tissue, the expression of these four markers and PD-L1 was consistently upregulated in pericystic fibrosis tissue (PD-L1: *P* = 0.048; all indicators *P* < 0.05, **Figure 2C**). After multiple comparison correction using the Benjamini-Hochberg method, the false discovery rate (*FDR*) increased to 0.216. Given the exploratory nature and small sample size of this pilot analysis, this outcome is expected; importantly, the upward trend and fluctuation amplitude of all markers were highly consistent, indicating the existence of a specific immune microenvironment with distinct structural features at the HAE invasive margin. The top 11 differentially expressed immune markers between paired CLT and DLT regions are summarized in **Supplementary Table 3**.

#### 3.1.2 Single-cell resolution profiling identifies key expanded cell subpopulations

To clarify the cellular origin of the observed overall polarization phenomenon at the single-cell level, we performed automated single-cell segmentation and unsupervised clustering analysis on the IMC datasets (**Figure 2D**). This analysis identified 19 distinct cell clusters and annotated them as three major lineages: myeloid, lymphoid, and stromal cells, based on their marker expression profiles.

In-depth phenotypic characterization analysis revealed two key cell subpopulations that selectively expanded in the CLT (**Figure 2E**): one was a “antigen-presenting cell (APC)-like” polarized neutrophil subpopulation expressing CD15^+^HLA-DR^+^PD-L1^+^ phenotype, and the other was an exhausted CD8^+^ T cell subpopulation (PD-1^+^TIM-3^+^LAG-3^+^, corresponding to 19 cluster). Consistent with the overall polarization results, the proportions of CD8^+^ T cells (clusters 3 and 19) and natural killer (NK) cells (clusters 17 and 18) were significantly higher in CLT than in DLT, while the proportion of B cells (clusters 5 and 7) was significantly lower.

#### 3.1.3 Correlation network analysis reveals coordinated immune checkpoint activation

We next examined co-expression relationships using Spearman correlations across the 6 paired CLT and DLT ROIs (**Figure 2F**). The main research results are summarized as follows:

Immune checkpoint axis: In CLT, PD-L1 and PD-1 exhibited a strong significant positive correlation (*ρ* = 0.89, *P* = 0.019). Significant synergistic expression was also observed between PD-L1 and other inhibitory ligands, including PD-L2 (*ρ* = 0.94, *P* = 0.005), indicating robust activation of inhibitory signaling pathways at the lesion margin.

T cell functional axis: CD8a expression showed significant positive correlations with both T-bet (*ρ* = 0.89, *P* = 0.019) and CD45RO (*ρ* = 0.83, *P* = 0.042), suggesting that CD8^+^ T cells in this region display a mixed effector-memory and exhausted phenotype.

Regional specificity: In contrast, the synergistic interactions among these immune checkpoint molecules were significantly attenuated in DLT, confirming that the immunosuppressive niche is specifically formed and maintained at the HAE invasive margin. Given the small number of ROIs (n=6 paired), these correlation findings are exploratory and warrant validation in larger cohorts.

### 3.2 scRNA-seq identifies a PD-L1^+^ neutrophil subpopulation with immunosuppressive features

**Figure 3:**
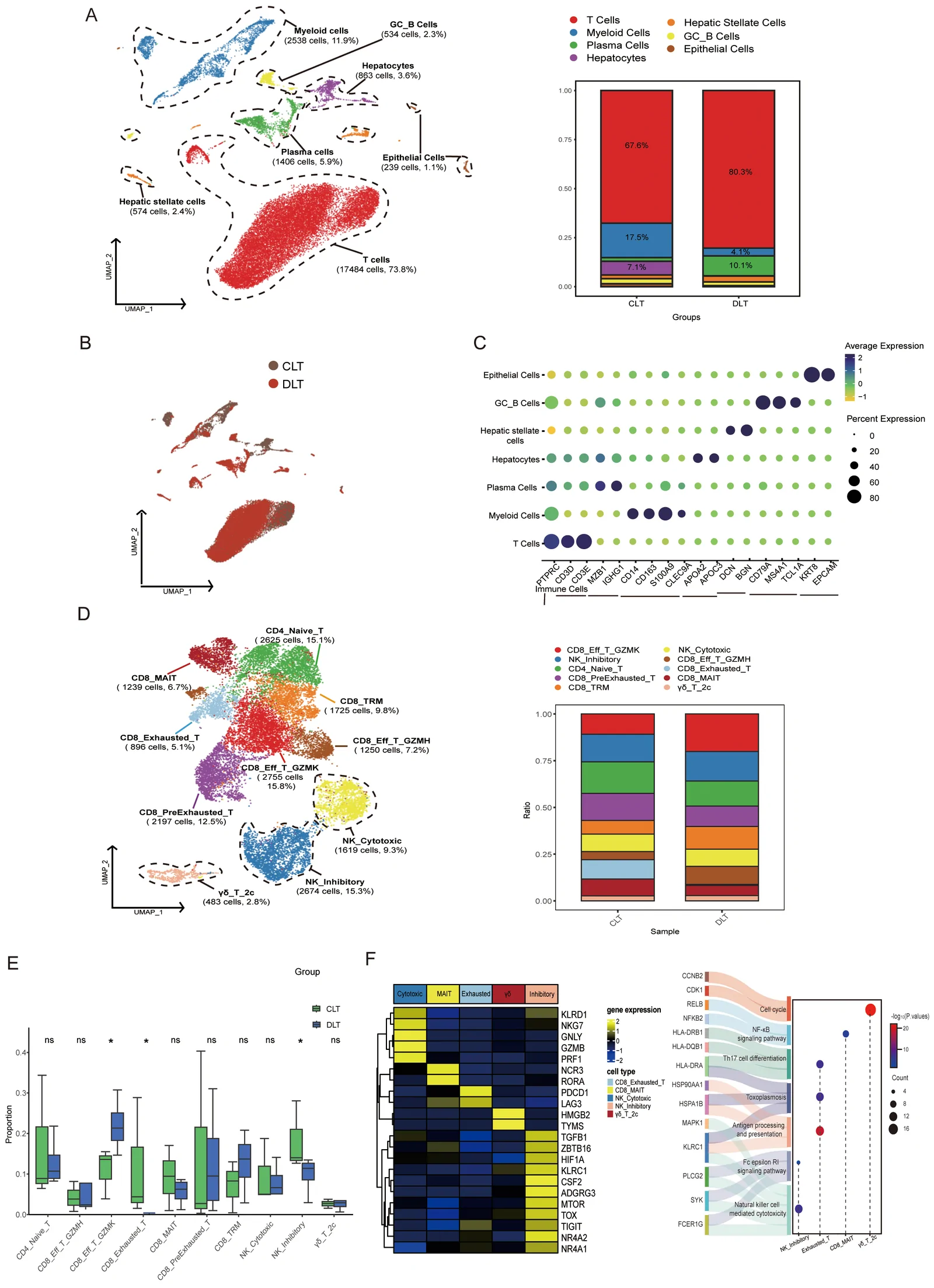
Single-cell transcriptomic landscape of immune cells in the invasive margin of hepatic alveolar echinococcosis. **(A)** UMAP visualization of all identified cell populations in HAE liver tissues (left) and the relative proportion of major cell lineages in CLT and DLT regions (right). The number and percentage of cells in each cell type are indicated. **(B)** UMAP plot showing the distribution of cells originating from CLT (brown) and DLT (red) regions. **(C)** Dot plot representing the average expression and percentage of marker genes used for major cell lineage identification. The size of dots indicates the percentage of cells expressing the gene, and the color intensity indicates the average expression level. **(D)** Sub-clustering of T cell and NK cell subsets (left) and the relative proportion of each subset in CLT and DLT samples (right). The number and percentage of cells in each subset are indicated. **(E)** Box plots showing the Statistical comparison of the proportions of T and NK cell subsets between paired CLT and DLT regions from 4 HAE patients. Data are presented as box plots; *P < 0.05, ns: not significant (paired Wilcoxon signed-rank test). **(F)** Heatmap showing the expression of key functional genes across different T/NK cell subsets (left), and a Sankey-Dot plot demonstrating the enriched signaling pathways (KEGG/GO) associated with specific cell types (right). The size of dots indicates the number of genes in each pathway, and the color intensity indicates the -log_10_(P value).

Leveraging 10x Genomics single-cell RNA sequencing (scRNA-seq), we next sought to cross-validate the spatial immune phenotypes identified by IMC at the transcriptomic level and further resolve the cellular heterogeneity of the HAE tissue microenvironment. Paired CLT and DLT specimens from four additional HAE patients were processed for library construction and deep sequencing. After stringent quality control filtering, a total of 48,236 high-quality single-cell transcriptomes were retained for downstream bioinformatic analysis (**Figure 3**). Detailed quality control metrics and global cell clustering profiles of the scRNA-seq dataset are summarized in **Supplementary Figure 2**.

#### 3.2.1 Remodeling of core immune lineages and CD8^+^ T cell exhaustion signatures

Unsupervised clustering of the combined dataset identified seven major immune and stromal cell lineages (**Figure 3A**). Grouped UMAP projections showed clear separation of cellular profiles between CLT and DLT samples, indicating substantial remodeling of the tissue microenvironment at the lesion margin (**Figure 3B**). Quantification of cell type frequencies revealed that myeloid cells (28.3% vs. 12.7%, *P* = 0.002) and T cells (37.6% vs. 18.9%, *P* = 0.004) were significantly enriched in CLT compared to matched DLT samples (**Figure 3C**).

High-resolution subclustering analysis further revealed that CD8^+^ T cells infiltrating CLT exhibited the classic exhausted transcriptional signature. Compared with CD8^+^ T cells isolated from DLT regions, CD8^+^ T cells from CLT coordinately upregulated multiple genes encoding inhibitory receptors (*PDCD1, LAG3, HAVCR2*) as well as the core transcription factor regulating T cell exhaustion, *TOX* (all adjusted *P* < 0.001, **Figures 3D, E**). These transcriptomic data independently verified the previously identified marked functional impairment of CD8^+^ T cells via IMC analysis. Signature gene expression profiles of distinct CD8^+^ T cell subsets are provided in **Supplementary Figure 4**. Differentially expressed markers between pre-exhausted and GZMK^+^ effector CD8^+^ T cells are listed in **Supplementary Table 4**.

#### 3.2.2 Suppressive polarization of “APC-like” PD-L1^+^ neutrophils

Consistent with our *in situ* IMC observations, myeloid cell subclustering analysis identified a unique subset of neutrophils that was selectively expanded in CLT. This subset not only highly expressed classical neutrophil markers (*S100A8, CXCR2*), but also significantly upregulated the immune checkpoint ligand gene *CD274* (encoding PD-L1 protein) as well as multiple core antigen-presenting genes (*HLA-DRA, HLA-DRB1*) (**Figure 3F**), consistent with the typical “APC-like” immunosuppressive phenotype. This finding provided crucial molecular evidence for the ability of this subset to mediate contact-dependent immunosuppression via the PD-L1/PD-1 axis. GO functional enrichment analysis results of PD-L1^+^ neutrophil subsets are shown in **Supplementary Figure 3**. Complete GO and KEGG enrichment analysis results are provided in **Supplementary Table 5**.

#### 3.2.3 Pseudotime and cell communication analyses confirm PD-L1/PD-1-mediated functional interactions

**Figure 4:**
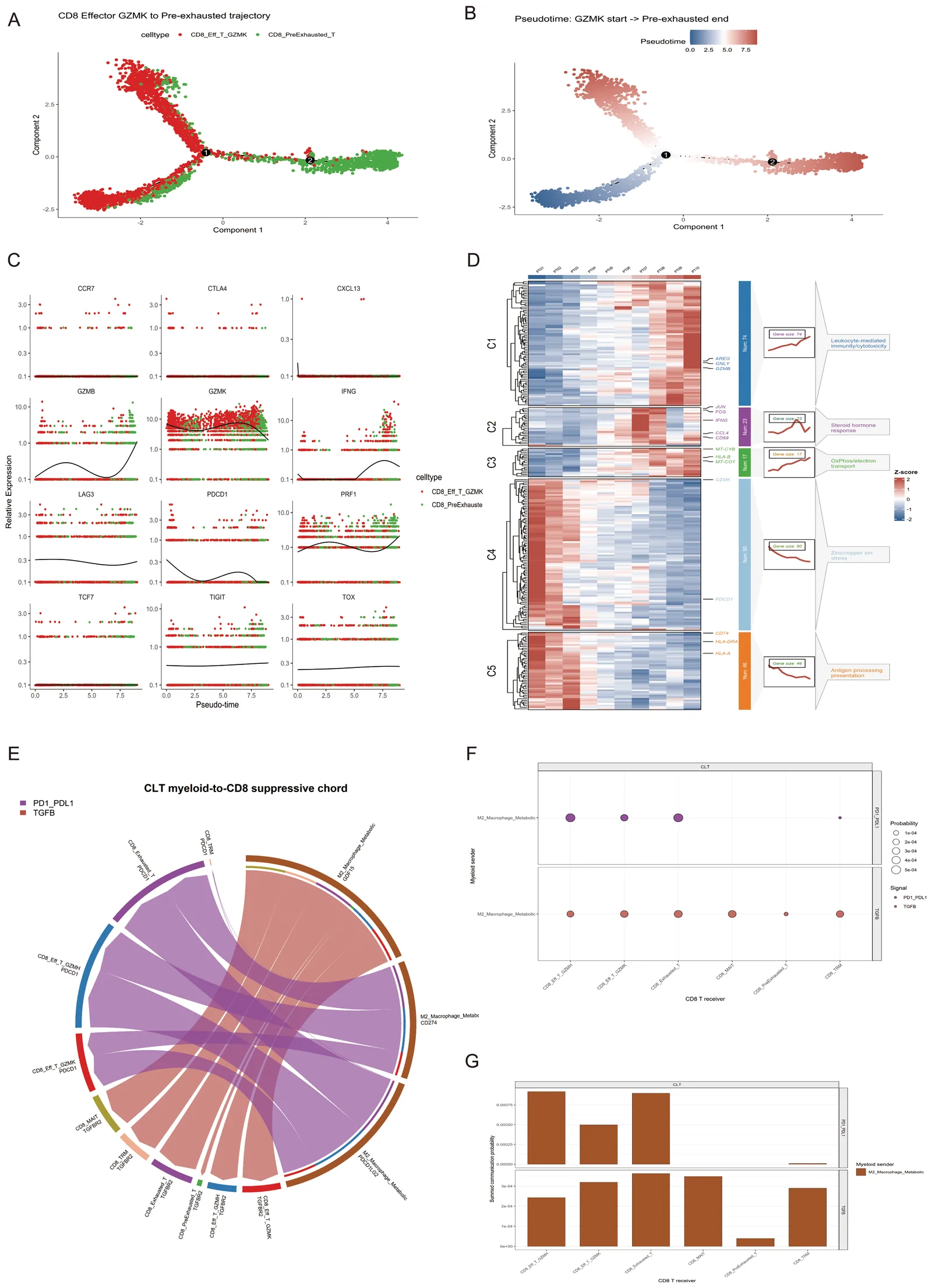
Trajectory analysis and cell-cell communication landscape of CD8^+^ T cells in HAE liver microenvironment. **(A)** Monocle pseudotime trajectory of the transition from CD8^+^ effector GZMK^+^ T cells to pre-exhausted T cells. Cells are colored by cell type. **(B)** Pseudotime distribution mapped across the differentiation axis, tracing a continuous transition from an early effector late pre-exhaustion (red). **(C)** Pseudotime-dependent expression kinetics for core effector and exhaustion markers (*GZMK, IFNG, PDCD1, TOX, TIGIT*). Individual cells are plotted as dots, overlaid with solid curves delineating smoothed expression trends. **(D)** Heatmap defining five gene co-expression modules (C1–C5) ordered along the differentiation axis. Color intensities reflect Z-transformed expression scores. Right: Key KEGG/GO pathways enriched within each respective module.**(E)** Chord plot mapping putative inhibitory cell-cell crosstalk within the CLT region, highlighting PD-1/PD-L1 and TGF-β signaling nodes between myeloid populations and CD8^+^ T cell subsets.**(F, G)** Dot **(F)** and bar **(G)**charts evaluating the probability and signaling strength of PD-1/PD-L1 and TGF-β interactions bridging specific myeloid senders and CD8^+^ T cell receivers. Dot size scales with interaction probability, while color depth corresponds to interaction magnitude. Statistical significance for interaction probabilities was derived via permutation testing.

Monocle 2 pseudotime analysis modeled the developmental trajectory of CD8^+^ T cells as a continuous transcriptomic arc from an effector-like state to terminal exhaustion (**Figures 4A, B**). Expression of effector genes *GZMB* and *IFNG* fell steadily along this path, coinciding with a rise in multiple inhibitory receptors (**Figures 4C, D**). When *in vitro*-generated “exhausted” CD8^+^ T cells were projected onto this trajectory, they localized almost entirely within the late-stage segment.

To explore the potential signal interactions between various immune cell subpopulations within CLT, this study further employed CellChat ligand receptor interaction analysis. The core highlighted results indicate that the PD-L1/PD-1 signaling axis is a key dominant pathway in this microenvironment (**Figures 4E, F**). Neutrophils with antigen-presenting cell like (APC like) phenotype characteristics are the main secretion source of PD-L1, while CD8^+^ T cells with transcriptome exhaustion characteristics are the receiving cells for this signal. It is worth noting that the probability of communication intensity between this group of cells in CLT is 5.8 times that of remote liver tissue (DLT) (*P* = 0.008; **Figure 4G**).

The above transcriptome patterns are highly consistent with the spatial close proximity observed by IMC between CD15^+^ neutrophils and CD8^+^ T cells, jointly confirming the existence of cell interactions that are co regulated by spatial location and molecular signals at the HAE invasion margin.

### 3.3 mIF independently validates the spatial colocalization of PD-L1^+^ neutrophils and exhausted CD8^+^ T cells

#### 3.3.1 Representative images reveal close spatial proximity between the two cell populations in the CLT

**Figure 5:**
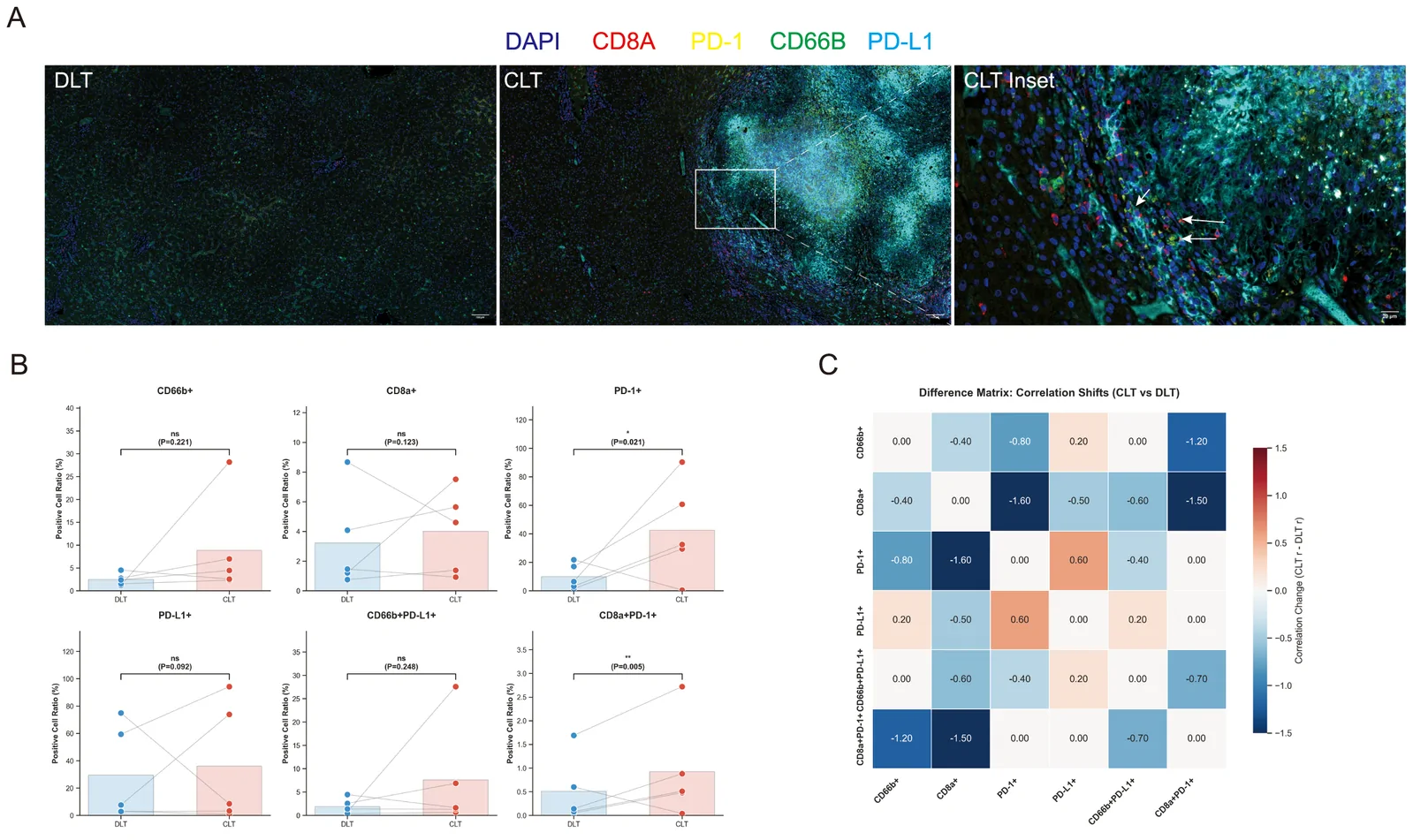
Multiplex immunofluorescence validation of spatial colocalization between PD-L1^+^ neutrophils and exhausted CD8^+^ T cells at the HAE invasive margin. **(A)** Representative multiple immunofluorescence (MIF) images from paired distal liver tissue (DLT) and collagen layer tissue (CLT) in patients with HAE. The illustration shows an enlarged view of the CLT area, and the white arrow indicates the close membrane to membrane contact between PD-L1^+^ neutrophils and PD-1^+^ CD8^+^ T cells. Main panels: 100 μm; inset (magnified region): 20 μm. **(B)** Quantitative analysis of immune cell subsets in paired DLT (blue dot) and CLT (red dot) tissues (n=5). The figure shows the percentage of CD66b^+^ neutrophils, CD8a^+^ T cells, PD-1^+^ cells, PD-L1^+^ cells, CD66b^+^PD-L1^+^ neutrophils, and CD8a^+^PD-1^+^ exhausted T cells are shown. The paired Wilcoxon signed rank test was used to calculate the p value. *P* < 0.05, ns: no significant difference. **(C)** Correlation shift Heatmap was used to compare the Spearman rank correlation coefficients of immune markers between CLT and DLT regions. Red indicates that the positive correlation in CLT increases, while blue indicates that the positive correlation in CLT decreases (or the negative correlation increases) compared with DLT.

Representative mIF images (**Figure 5A**) showed high-density infiltration of CD66b+ neutrophils and CD8a^+^ T cells in the pericystic fibrosis tissue (CLT). Some neutrophils show PD-L1 positive expression, while a certain proportion of CD8 ^+^ T cells express PD-1.

The most prominent feature is that PD-L1^+^ neutrophils and PD-1^+^ CD8^+^ T cells are frequently distributed in close membrane-to-membrane proximity (indicated by white arrows). This spatial colocalization pattern supports the potential for direct physical interaction between the two cell populations, although proximity alone does not provide definitive evidence of direct PD-1/PD-L1 molecular binding at single-molecule resolution. In the distal liver parenchyma tissue (DLT), immune cell infiltration is extremely sparse, and no obvious co-localization phenomenon was observed (scale bar: 100 μm).

#### 3.3.2 Quantitative analysis confirms the CLT-specific enrichment of key immune subpopulations

Quantitative analysis based on the paired Wilcoxon signed-rank test (**Figure 5B**) demonstrated that total PD-1^+^ cells (46.93 ± 12.70% vs. 5.74 ± 3.00%, *P* = 0.022) and CD8a^+^PD-1^+^ exhausted T cells (1.04 ± 0.43% vs. 0.40 ± 0.32%, *P* = 0.005) were significantly enriched in CLT. Although limited by the small sample size, the frequency of CD66b^+^PD-L1^+^ immunosuppressive neutrophils showed a consistent but statistically non-significant increasing trend in CLT (*P* = 0.249), with an approximately 6.1-fold elevation relative to DLT, and was consistently upregulated in four of five enrolled patients.

#### 3.3.3 Correlation analysis supports the existence of a localized immunosuppressive network

Spearman rank correlation analysis with exact permutation testing (**Figure 5C**) showed that, in CLT, PD-L1 expression was positively correlated with PD-1 (*ρ* = 0.70, *P* = 0.188), and CD66b expression was positively correlated with PD-L1 (*ρ* = 0.70, *P* = 0.188), suggesting that neutrophils serve as the primary cellular source of PD-L1 at the invasive margin. In comparison, these correlation relationships were markedly attenuated in DLT. These findings were highly consistent with our previous IMC and scRNA-seq results, further validating the spatial co-localization of PD-L1^+^ neutrophils and exhausted CD8^+^ T cells at the molecular correlation level.

### 3.4 Neutrophils mediate contact-dependent CD8^+^ T cell exhaustion via the PD-L1/PD-1 axis

**Figure 6:**
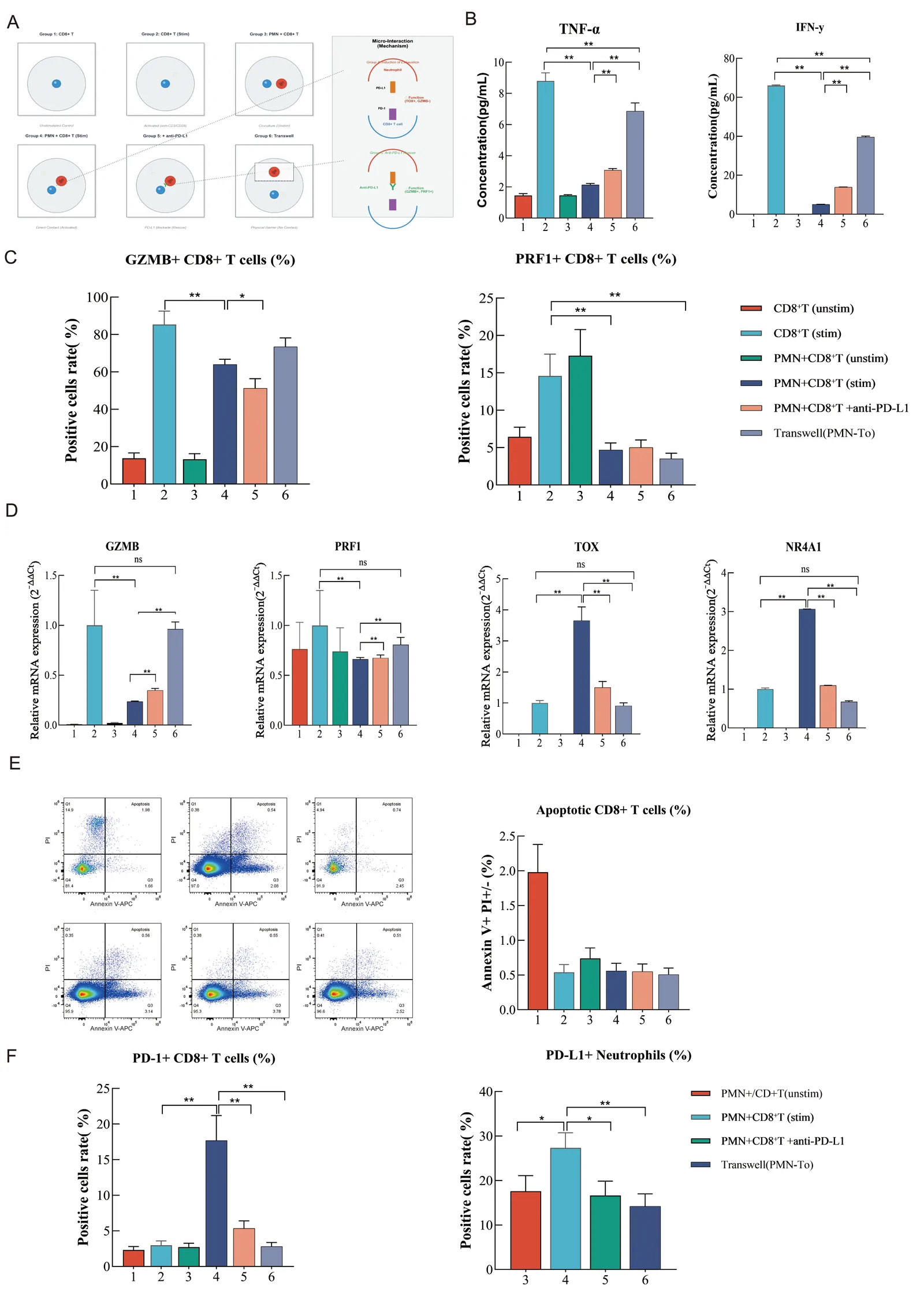
PMN-mediated contact-dependent suppression of CD8^+^ T cell effector function in an *in vitro* co-culture model. **(A)** Schematic diagram of experimental design. Six groups were set up to study the interaction between PMN (polymorphonuclear neutrophils) and CD8^+^ T cells, including Transwell experiment to determine whether physical contact is required, and anti-PD-L1 antibody treatment group to verify PD-1/PD-L1 checkpoint axis. The right panel showed the mechanism of contact dependent immunosuppression. **(B)** The concentrations of TNF-α and IFN-γ in the supernatant of six experimental groups were analyzed by ELISA. **(C)** Frequencies of GZMB^+^ and PRF1^+^ CD8^+^ T cells in the six groups, as determined by flow cytometry. **(D)** RT-qPCR analysis of *GZMB, PRF1, TOX*, and *NR4A1* mRNA expression levels in CD8^+^ T cells across the experimental groups. Data are presented as relative mRNA expression normalized to the control group (Group 2). **(E)** Representative flow cytometry plots (left) and quantification (right) of apoptotic CD8^+^ T cells (Annexin V^+^ PI^+^/-) in the co-culture system. **(F)** Frequencies of PD-1^+^ CD8^+^ T cells and PD-L1^+^ neutrophils (PMNs) across the relevant experimental conditions (Groups 3-6). Statistical Analysis: Data are represented as mean ± SEM (n = 3 independent experiments). Significant differences were determined by one-way ANOVA followed by Tukey’s *post-hoc* test. *P < 0.05, **P < 0.01, ns: not significant.

To elucidate the functional mechanisms underlying the spatial interaction between polarized neutrophils and CD8^+^ T cells at the HAE margin, we established an *in vitro* co-culture system using peripheral blood neutrophils isolated from HAE patients and CD8^+^ T cells from healthy volunteers at a 2:1 neutrophil-to-T cell ratio (**Figure 6A**).

#### 3.4.1 Neutrophils specifically inhibit the effector functions of activated CD8^+^ T cells

Following 72 h of co-culture with purified neutrophils, CD8^+^ T cells activated with CD3/CD28 magnetic beads exhibited a profound decline in effector cytokine production: IFN-γ secretion dropped to 7.6% and TNF-α to 24.2% of the levels detected in activated CD8^+^ T cells cultured alone (both *P* < 0.001, **Figure 6B**). In contrast, neutrophils exerted no significant effect on cytokine secretion by resting CD8^+^ T cells (both *P* > 0.05, **Figure 6B**).

Intracellular flow cytometric staining showed a pronounced decrease in GZMB protein abundance (*P* < 0.05, **Figure 6C**). Consistent with the protein-level findings, RT-qPCR verification revealed that *GZMB* and *PRF1* transcript levels decreased to 0.24-fold and 0.66-fold of control levels, respectively (both *P* < 0.01, **Figure 6D**). Annexin V/PI dual staining further confirmed that co-culture did not induce a significant elevation in the apoptosis rate of CD8^+^ T cells (*P* > 0.05, **Figure 6E**). Taken together, these results demonstrate that neutrophils drive CD8^+^ T cell dysfunction primarily by suppressing effector function rather than promoting cell apoptosis.

#### 3.4.2 Neutrophils mediate the suppressive effect via the PD-L1/PD-1 axis

After 72 hours of co-culture, we found coordinated increases of PD-1 and PD-L1 on the two cell types. The surface PD-L1 level on neutrophils increased 1.6 times, and PD-1 expression on CD8^+^ T cells rose 5.9 times (*P* < 0.001, **Figure 6F**). To clarify the role of this signal pathway in immune suppression, we added 10 μg/mL anti-PD-L1 neutralizing antibody into the culture medium.

Neutralization of PD-L1 significantly restored the secretion of IFN-γ and TNF-α by CD8^+^ T cells, and concurrently downregulated the mRNA levels of exhaustion-related transcription factors *TOX* and *NR4A1* (all *P* < 0.05, **Figures 6B, D**). Overall, these functional data indicate that the PD-1/PD-L1 axis serves as an important functional mediator underlying neutrophil-mediated suppression of CD8^+^ T cell effector function in this *in vitro* model.

#### 3.4.3 The suppressive effect is highly dependent on direct cell-to-cell physical contact

The use of a Transwell physical separation system (0.4 μm pore size, allowing only soluble factor exchange) almost completely abolished the neutrophil-mediated suppression of CD8^+^ T cells. Compared with the direct neutrophil-CD8^+^ T cell co-culture control, IFN-γ secretion was restored to 60% of that observed in the activated T cell alone group, and transcript levels of *TOX* and *NR4A1* returned to near-baseline values (**Figures 6B, D**). These results confirm that the immunosuppressive effect of PD-L1^+^ neutrophils on CD8^+^ T cells is predominantly mediated by direct cell-to-cell contact.

We further evaluated the contribution of the PD-L1/PD-1 axis via antibody neutralization. Relative to the direct co-culture control, anti-PD-L1 treatment also significantly reversed CD8^+^ T cell suppression, restoring IFN-γ secretion to 21.1% of the activated T cell alone level. Of note, the Transwell separation and anti-PD-L1 blockade were performed under distinct experimental systems to address separate mechanistic questions, and thus direct head-to-head quantitative comparison between the two groups is not appropriate. The finding that single PD-L1 blockade only partially reversed contact-dependent suppression suggests the existence of additional contact-dependent immunosuppressive mechanisms beyond the PD-L1/PD-1 axis, such as other inhibitory ligand-receptor pairs (e.g., TIGIT-CD155, LAG-3-MHC class II), which warrant further targeted investigation.

In summary, neutrophils derived from HAE patients can induce CD8^+^ T cell exhaustion in a contact-dependent manner, in which the PD-L1/PD-1 axis serves as an important functional mediator. These findings provide direct experimental evidence for the mechanisms of immune evasion in HAE. Representative flow cytometry results of *in vitro* co-culture and Transwell assays are shown in **Supplementary Figure 5**.

### 3.5 *In vivo* validation: targeting neutrophils or PD-L1 ameliorates hepatic pathology and reverses CD8^+^ T cell exhaustion

#### 3.5.1 Targeted interventions significantly reduce parasite burden and ameliorate hepatic pathology

**Figure 7:**
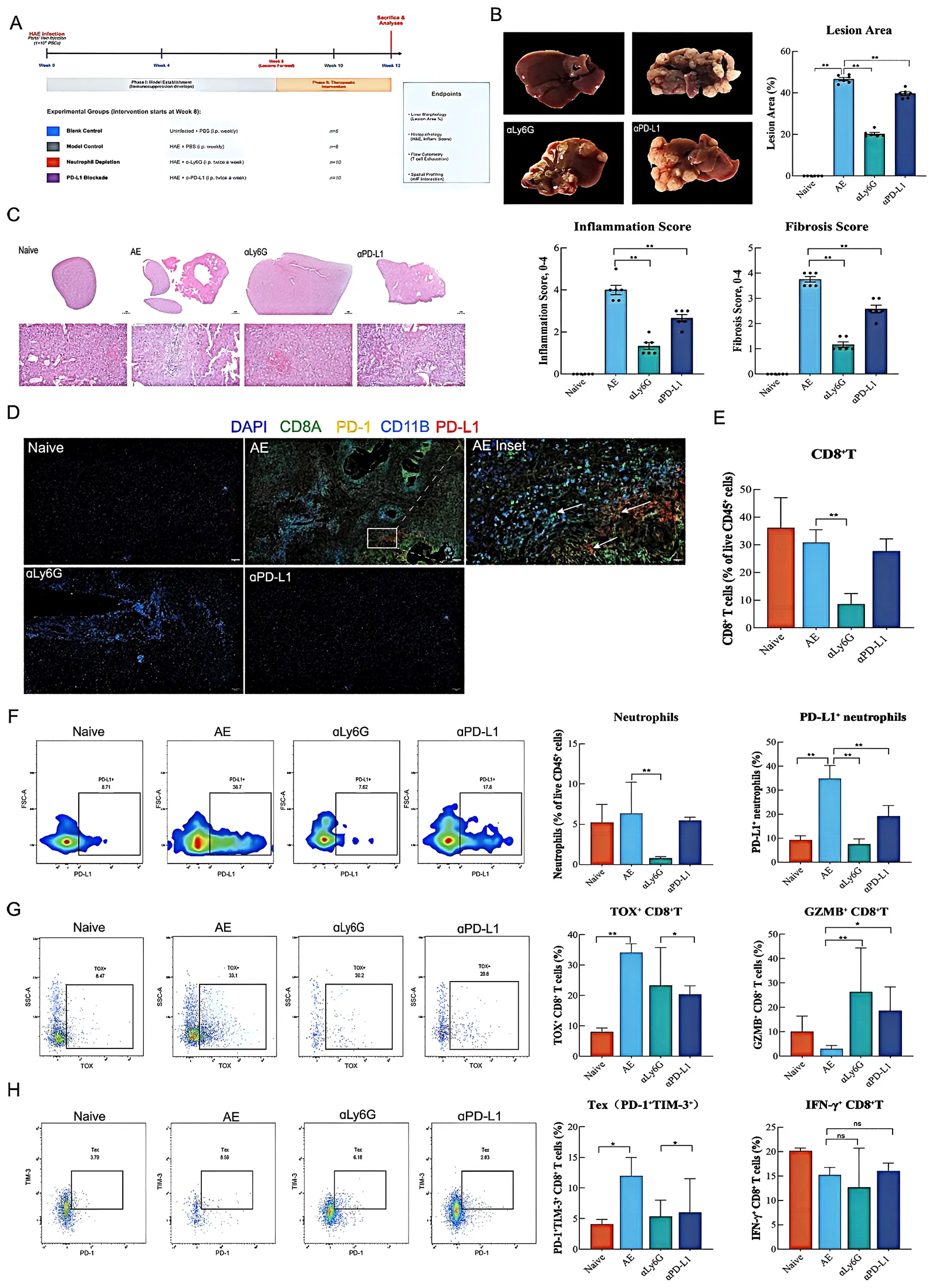
*In vivo* targeting of neutrophils or PD-L1 ameliorates hepatic pathology and reverses CD8^+^ T cell exhaustion. **(A)** Experimental design of the HAE mouse model and therapeutic interventions. **(B)** Representative macroscopic images of liver morphology and quantitative analysis of the lesion area across different groups. **(C)** Representative H&E staining images of liver tissues (left) and semi-quantitative scoring of hepatic inflammation and fibrosis (right). **(D)** Representative multiplex immunofluorescence (mIF) images of the invasive margin. Blue: DAPI; Green: CD8A; Yellow: PD-1; Cyan: CD11B; Red: PD-L1. The Inset highlights direct physical contact between myeloid cells and CD8^+^ T cells. **(E)** Quantitative analysis of the absolute frequency of total CD8^+^ T cells in the liver. **(F)** Representative flow cytometry plots and frequency analysis of total neutrophils and PD-L1^+^ neutrophils among viable intrahepatic CD45^+^ cells. **(G)** Flow cytometry analysis of TOX^+^ CD8^+^ T cells and GZMB^+^ CD8^+^ T cells, reflecting the exhaustion status and effector function of T cells, respectively. **(H)** Representative flow plots and quantitative analysis of terminally exhausted CD8^+^ T cells (defined as PD-1^+^TIM-3^+^) and IFN-γ^+^ CD8^+^ T cells. Statistical Analysis: Data are presented as mean ± SEM (n=6–10 mice per group). Significant differences were assessed using one-way ANOVA followed by Tukey’s *post-hoc* test. *P < 0.05, **P < 0.01, ns: not significant.

To validate the hypothesis that PD-L1^+^ neutrophils mediate CD8^+^ T cell exhaustion *in vivo*, we established a delayed-treatment C57BL/6 mouse model of HAE (interventions commenced at 8 weeks post-infection and continued for 4 weeks, as described in Methods 2.5) and evaluated the therapeutic efficacy of α-Ly6G (neutrophil depletion) and α-PD-L1 (checkpoint blockade) (**Figure 7A**). Macroscopic observation and quantitative analysis showed that the livers of the AE model group were covered with grayish-white nodular lesions (lesion area proportion 46.65 ± 0.79%); in contrast, targeted interventions significantly reduced lesion volumes. Specifically, the lesion proportions in the α-Ly6G and α-PD-L1 groups significantly decreased to 20.27 ± 0.65% and 39.66 ± 0.88%, respectively (**Figure 7B**, *P* < 0.001 vs. AE). Furthermore, H&E staining confirmed that both interventions significantly alleviated hepatic inflammatory infiltration and bridging fibrosis (**Figure 7C**, *P* < 0.001). Detailed quantitative data of liver lesion burden and immune cell counts in mouse models are summarized in **Supplementary Table 7**.

#### 3.5.2 Multiplex immunofluorescence reveals an *in situ* contact-dependent immunosuppressive niche

To investigate the spatial basis for the therapeutic effects observed in our animal studies, we performed mIF staining on lesion margin tissues from all experimental groups (**Figure 7D**). In the untreated AE model group, we observed the formation of a dense immunosuppressive niche at the lesion edge, with prominent accumulation of CD11b^+^ myeloid cells and CD8^+^ T cells. At a higher magnification (**Figure 7E**), we observed extensive close membrane-to-membrane contacts between PD-L1^+^ myeloid cells and PD-1^+^ CD8^+^ T cells. These observations provided direct morphological evidence for the existence of contact-dependent immunosuppression within this microenvironment.

Both therapeutic interventions significantly disrupted this organized spatial immune network. In the ɑ-Ly6G neutrophil clearance group, CD11b^+^ myeloid cells and related PD-L1/PD-1 signals at the edge of the lesion were almost completely eliminated. In contrast, the ɑ-PD-L1 blockade group retained most of the CD11b^+^ myeloid cells in the marginal region, but showed a significant reduction in the co-localization of PD-L1 and PD-1 signals.

These findings confirmed that both intervention strategies physically disrupted intercellular inhibitory interactions sustaining the immunosuppressive microenvironment at the HAE invasive margin.

#### 3.5.3 Clearance of the myeloid barrier reverses T cell exhaustion and reveals a “homing-function” paradox

Combined with flow cytometry, we further quantified the remodeling patterns of microenvironmental cell populations. In the AE group, the proportion of PD-L1^+^ cells among viable CD45^+^ neutrophils was significantly increased. α-Ly6G treatment achieved massive neutrophil depletion, leading to near-total elimination of the PD-L1^+^ subpopulation; the frequency of this suppressive population also significantly decreased in the α-PD-L1 group (**Figure 7F**), indicating that both strategies effectively weakened the suppressive myeloid barrier.

Subpopulation analysis of CD8^+^ T cells further revealed a distinct “homing-function” paradox: Homing restriction: Total CD8^+^ T cell infiltration in the α-Ly6G group was significantly reduced (**Figure 7E**, as quantified in **Supplementary Figure 6**), suggesting that neutrophils play a crucial role in directing T cell homing; no significant change was observed in the α-PD-L1 group.

Effector recovery and exhaustion reversal: Despite the reduced number of infiltrating T cells, both interventions significantly restored T cell cytotoxicity, with substantial upregulation of GZMB^+^ and IFN-γ^+^ cell proportions (**Figures 7G, H**, GZMB expression increased 8.6-fold in α-Ly6G group and 6.1-fold in α-PD-L1 group compared to AE). Additionally, the proportions of TOX^+^ cells (**Figure 7G**) and terminally exhausted T cells (Tex^+^, PD-1^+^TIM-3^+^) (**Figure 7H**) significantly decreased in the α-PD-L1 group. Although the Tex^+^ proportion slightly increased in the α-Ly6G group (**Figure 7H**), this was accompanied by a robust recovery of GZMB^+^ cells. This likely reflects transient co-expression of inhibitory receptors on recently reactivated effector T cells rather than a state of terminal exhaustion, as previously observed following checkpoint therapy (18).

**Figure 8:**
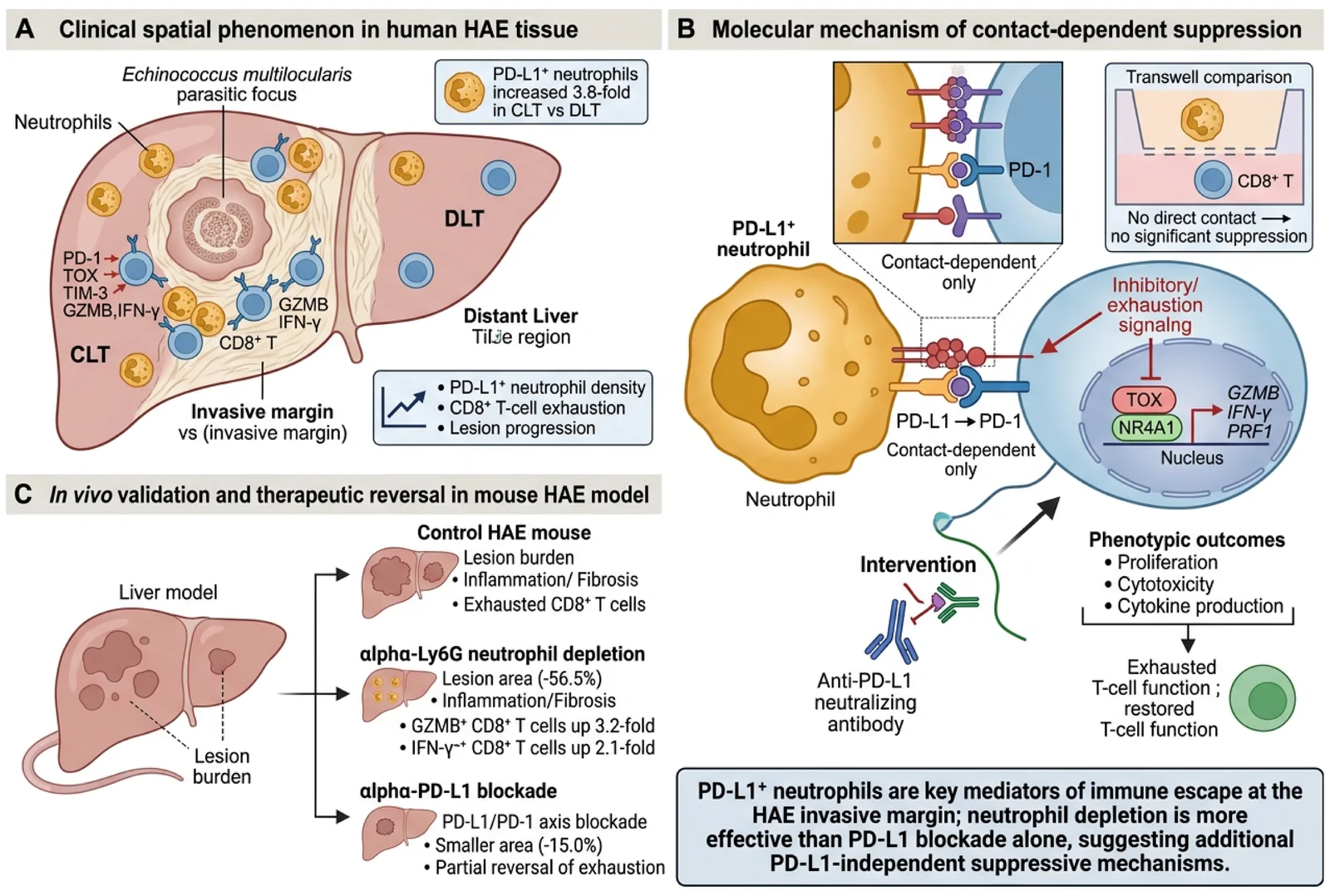
Graphical Abstract. PD-L1^+^ neutrophils mediate contact-dependent CD8^+^ T cell exhaustion at the invasive margin of hepatic alveolar echinococcosis. **(A)** Clinical spatial phenomenon in human HAE tissues. PD-L1^+^ neutrophils are significantly enriched at the invasive margin (CLT) compared with distal liver tissue (DLT), and their density is positively correlated with CD8^+^ T cell exhaustion and lesion progression. **(B)** Molecular mechanism of contact-dependent immune suppression. PD-L1^+^ neutrophils directly interact with PD-1 on CD8^+^ T cells via cell-cell contact, activating the downstream TOX/NR4A1 signaling axis and inhibiting the expression of effector molecules. This inhibitory effect was eliminated by anti-PD-L1 antibody treatment. **(C)** *In vivo* validation and therapeutic reversal in HAE mouse model. Neutrophil clearance (α-Ly6G) significantly reduced the lesion load and restored the function of CD8^+^ T cells, showing a better therapeutic effect than blocking PD-L1 alone, suggesting that there are other inhibition mechanisms independent of PD-L1.

In conclusion, the *in vivo* and *in situ* data were mutually consistent, indicating that the contact-dependent immunosuppressive barrier centered on PD-L1^+^ neutrophils constitutes the core mechanism driving local immune escape at the HAE invasive margin. Disruption of this cellular network restores the effector function of CD8^+^ T cells, thereby facilitating effective regression of parasitic lesions. A schematic model summarizing the core mechanism identified in this study is shown in **Figure 8**.

## 4 Discussion

This study integrated imaging mass cytometry, single-cell RNA sequencing, multiplex immunofluorescence, *in vitro* functional co-culture assays and *in vivo* therapeutic intervention to systematically dissect the cellular and molecular basis of localized CD8^+^ T cell exhaustion at the invasive margin of hepatic alveolar echinococcosis. The core finding is that APC-like PD-L1^+^ neutrophils selectively enriched at the lesion collagenous layer contribute to CD8^+^ T cell exhaustion in a contact-dependent manner, and that disruption of this myeloid-T cell crosstalk effectively restores anti-parasitic immunity and reduces lesion burden. These findings extend the current understanding of HAE immune evasion mechanisms and provide a preclinical rationale for myeloid-targeted immunotherapy.

### 4.1 Spatial enrichment of PD-L1^+^ neutrophils at the lesion invasive margin

Previous studies have documented widespread CD8^+^ T cell dysfunction and upregulation of multiple co-inhibitory receptors in HAE lesions, but the specific cellular subsets driving localized immunosuppression at the parasite-host interface have not been clearly delineated (11, 12). Earlier work mainly attributed myeloid-mediated immunosuppression to myeloid-derived suppressor cells (MDSCs) in HAE (17). In the present study, high-resolution spatial profiling further refined this concept by identifying a distinct subset of APC-like PD-L1^+^ neutrophils that specifically accumulate at the invasive margin. This subset co-expresses classical neutrophil markers (S100A8, CXCR2) along with antigen-presenting molecules (HLA-DRA, HLA-DRB1) and the inhibitory ligand PD-L1, resembling the PMN-MDSC phenotype described in chronic infection and tumor settings (13, 15). Similar PD-L1-expressing suppressive neutrophils have also been validated in other chronic parasitic infectious models such as visceral leishmaniasis (19, 20), further supporting the conserved immunosuppressive phenotype of neutrophils under persistent pathogen stimulation.

Notably, the enrichment of PD-L1^+^ myeloid cells at the invasive front shows both parallels and distinctions from solid tumor microenvironments. In tumors, neutrophil recruitment and PD-L1 polarization are primarily driven by tumor-derived chemokines and oncogenic signals (14); in HAE, this process is likely triggered by parasite-derived antigens, stromal fibrotic cues and local inflammatory mediators such as IL-17A, representing a distinct regulatory paradigm of chronic helminth-induced immunosuppression. The strong positive correlation between neutrophil density and local IL-17A levels observed in our IMC data provides preliminary support for this hypothesis, but further targeted functional validation is required.

### 4.2 Contact-dependent CD8^+^ T cell suppression mediated by PD-L1^+^ neutrophils

Functional validation via *in vitro* co-culture assays confirmed that PD-L1^+^ neutrophils suppress CD8^+^ T cell effector function primarily through direct cell-cell contact rather than soluble mediators. Transwell physical separation largely abrogated the suppressive effect, restoring IFN-γ secretion and downregulating exhaustion-associated transcription factors TOX and NR4A1, which establishes contact dependence as the dominant mode of neutrophil-mediated T cell inhibition in this system (21). Neutralization of PD-L1 significantly reversed T cell suppression, indicating that the PD-L1/PD-1 axis serves as an important functional mediator of this contact-dependent inhibition.

It should be emphasized that the Transwell system and anti-PD-L1 antibody treatment represent distinct experimental systems addressing separate mechanistic layers: Transwell simultaneously eliminates all direct cell-cell interactions and alters the diffusion kinetics of soluble factors, whereas anti-PD-L1 antibody targets only the single PD-L1/PD-1 signaling axis. Direct head-to-head quantitative comparison between the two groups is therefore methodologically inappropriate. The observation that single PD-L1 blockade only partially reversed contact-dependent suppression suggests the existence of additional contact-dependent inhibitory pathways beyond PD-L1/PD-1. Candidate molecules include TIGIT-CD155, LAG-3-MHC class II and other co-inhibitory ligand-receptors, which synergistically lock T cells into terminal exhaustion together with PD-1 (22, 23, 25, 26). Multiple helminth infection models have reported similar multi-checkpoint co-expression patterns on exhausted T cells (27, 28), consistent with the multi-inhibitory signature detected in our spatial transcriptomic data, further supporting the rationale for combinatorial checkpoint blockade in HAE (12, 24, 31).

### 4.3 *In vivo* therapeutic efficacy and multi-mechanistic interpretation

In the delayed-treatment HAE mouse model, both neutrophil depletion and PD-L1 blockade significantly reduced parasite burden, alleviated hepatic inflammation and fibrosis, and restored CD8^+^ T cell cytotoxic function. Therapeutic intervention was initiated at 8 weeks post-infection, when lesions have progressed to the chronic fibrotic stage with established immunosuppression, mimicking the clinical scenario in which HAE patients are typically diagnosed at an advanced chronic phase (4, 17). This delayed treatment design therefore has greater clinical translational relevance than prophylactic early intervention.

Notably, neutrophil depletion achieved superior therapeutic efficacy compared with single-agent PD-L1 blockade. This efficacy gap cannot be attributed solely to elimination of the PD-L1/PD-1 axis, and reflects the broader biological effects of global neutrophil elimination. Beyond removing PD-L1^+^ suppressive neutrophils, α-Ly6G treatment also eliminates neutrophil-derived pro-fibrotic mediators, reactive oxygen species, neutrophil extracellular traps (NETs) and other membrane-bound inhibitory ligands, all of which contribute to lesion progression and T cell dysfunction (13, 15, 16). The superior *in vivo* efficacy therefore reflects disruption of the entire myeloid suppressive barrier, not just blockade of a single checkpoint pathway. This multi-layered regulatory mode of myeloid cells aligns with metabolic immune regulation patterns identified in chronic liver inflammatory diseases such as hepatitis B virus infection (29), which shares a similar fibrotic suppressive microenvironment with HAE. This mechanistic distinction has important translational implications: for advanced HAE with extensive fibrotic lesions, strategies targeting the entire myeloid suppressive compartment may yield better outcomes than single checkpoint inhibition alone.

### 4.4 The “homing-function paradox” of neutrophils in HAE

A particularly notable finding of this study is the “homing-function paradox” of neutrophils in HAE: neutrophils act as key chemotactic mediators that recruit CD8^+^ T cells to the lesion site, but once T cells arrive, neutrophils impose contact-dependent suppression that abrogates their effector function. Our scRNA-seq data provide transcriptional support for this recruitment effect: neutrophils at the invasive margin highly express T cell-attracting chemokines including CXCL9, CXCL10 and CCL5, whose cognate receptors CXCR3 and CCR5 are abundantly expressed on CD8^+^ T cells. Chemokine-mediated leukocyte trafficking regulated by neutrophils has been widely validated across tumor and chronic infection models (13, 14, 30).

This paradox carries important conceptual implications: simply increasing CD8^+^ T cell infiltration does not guarantee improved parasite control, as recruited T cells become functionally silenced by the local neutrophil-dominated suppressive niche. Restoring the functional competence of tissue-resident T cells may be at least as important as expanding T cell numbers for effective control of HAE. It should be acknowledged that the chemokine expression data are currently validated only at the transcriptomic level; protein-level quantification and functional neutralization assays are required to definitively establish the mechanistic basis of neutrophil-dependent T cell recruitment.

### 4.5 Limitations and future perspectives

Several limitations of this study should be acknowledged.

First, the patient cohort is relatively small and single-center. Age stratification and subgroup analysis of preoperative albendazole treatment were not performed due to limited sample size. Larger multicenter prospective cohorts are needed to validate the prognostic value of PD-L1^+^ neutrophil density and the generalizability of the spatial immune niche phenotype.

Second, the intrahepatic protoscolex injection mouse model, while widely used for standardized preclinical therapeutic evaluation in HAE research (17, 18), differs from the natural oral infection route of human disease and cannot fully recapitulate the process of oncosphere migration and liver colonization. This model was chosen for its high success rate, uniform lesion progression and low inter-individual variability, which are critical for reliable therapeutic comparison. The inoculum dose of 1×10^4^ protoscoleces is consistent with multiple published dose-gradient studies and reliably induces chronic fibrotic lesions by week 8. Follow-up validation in oral infection models will be important to confirm the translational relevance of the findings.

Third, we only performed qualitative macroscopic assessment of liver lesions at week 8 before treatment initiation, without systematic quantitative baseline data on parasite burden, pathological scores or immune cell profiles at this pre-intervention time point. Although the uniform disease phenotype in the terminal untreated AE group supports comparable baseline status across all infected mice, the absence of pre-treatment quantitative data precludes definitive distinction between reversal of established disease and prevention of further progression. Longitudinal sampling at multiple disease time points will be included in future studies.

Fourth, the *in vitro* co-culture assay did not include an isotype-matched IgG control for the anti-PD-L1 neutralizing antibody. Pre-experiments confirmed that the PBS solvent had no significant effect on T cell cytokine secretion, and the isotype group was omitted to conserve scarce primary immune cells from HAE patients. Potential Fc-mediated non-specific effects on T cell function therefore cannot be fully excluded. For *in vivo* experiments, isotype-matched rat IgG2a/IgG2b were validated in pre-tests and showed no significant non-specific effects on lesions, hepatic pathology or T cell function, supporting the reliability of the *in vivo* control design. Subsequent *in vitro* validation experiments using sufficient healthy donor cells will supplement isotype control groups.

Fifth, spatial membrane-to-membrane proximity observed by IMC and mIF indicates the potential for direct interaction but does not constitute definitive evidence of direct PD-1/PD-L1 molecular binding at single-molecule resolution. Proximity ligation assay (PLA) or super-resolution confocal microscopy will be applied in follow-up studies to directly visualize receptor-ligand interaction *in situ*.

Sixth, the current *in vivo* experiments cannot unequivocally attribute the therapeutic effects exclusively to neutrophil-derived PD-L1. Systemic anti-PD-L1 blockade targets PD-L1 on all immune, stromal and parenchymal cells, and global neutrophil depletion exerts broader anti-inflammatory and anti-fibrotic effects beyond elimination of PD-L1-expressing neutrophils. The contribution of PD-L1 from other cell types therefore cannot be completely ruled out. Neutrophil-specific PD-L1 conditional knockout mice (Ly6G-Cre/MRP8-Cre × Cd274^fl/fl^) and adoptive transfer experiments using wild-type versus PD-L1-deficient neutrophils will be performed in future work to establish definitive cell-type-specific causality.

Seventh, the chemokine axis mediating neutrophil-dependent CD8^+^ T cell recruitment has only been characterized at the transcriptomic level. Functional validation via neutralization assays and protein-level quantification of CXCL9, CXCL10 and CCL5 in the lesion microenvironment are required to confirm their mechanistic role in T cell homing.

Eighth, CD8^+^ T cells used in the *in vitro* co-culture system were isolated from healthy donors rather than HAE patients, to ensure a uniform functional baseline and avoid confounding by pre-existing T cell exhaustion. While this is a widely accepted design in neutrophil immunoregulation research, it cannot fully replicate the autologous immune microenvironment in patients. Autologous co-culture experiments using patient-derived T cells will be performed to further confirm the clinical relevance of the findings.

### 4.6 Conclusion

In conclusion, this study identifies a spatially defined immunosuppressive niche at the HAE invasive margin, where APC-like PD-L1^+^ neutrophils contribute importantly to localized CD8^+^ T cell exhaustion via direct cell-cell contact. The PD-L1/PD-1 axis acts as a key functional mediator of this process, and multiple synergistic co-inhibitory ligand-receptor pairs such as LAG-3 and TIGIT also participate in contact-dependent immune suppression (22, 25, 26, 31). Targeted neutrophil depletion disrupts the entire myeloid suppressive barrier and achieves superior therapeutic efficacy compared with single PD-L1 blockade, supporting the concept that targeting myeloid suppressor cells represents a promising immunotherapeutic strategy for chronic HAE. Collectively, these data enrich the mechanistic understanding of helminth immune evasion conserved across multiple parasitic infections (19, 20, 27, 28) and provide a preclinical foundation for developing precision immunotherapy for hepatic alveolar echinococcosis.

## Data Availability

The single-cell RNA‑sequencing data generated in this study have been deposited in the Genome Sequence Archive (GSA) at the National Genomics Data Center (NGDC), China National Center for Bioinformation / Beijing Institute of Genomics, Chinese Academy of Sciences, under the project accession number HRA032777. The data are publicly accessible at: https://ngdc.cncb.ac.cn/gsub/submit/bioproject/list.
