# Supplementary material for: PD-L1+ neutrophils at the invasive margin drive contact-dependent CD8+ T cell exhaustion in hepatic alveolar echinococcosis
Source: Front Immunol. 2026 Sep 10;17:1917606. doi: 10.3389/fimmu.2026.1917606 (PMC13600833; doi:10.3389/fimmu.2026.1917606)
Supplement: Supplementary file 1 [file DataSheet1.pdf]

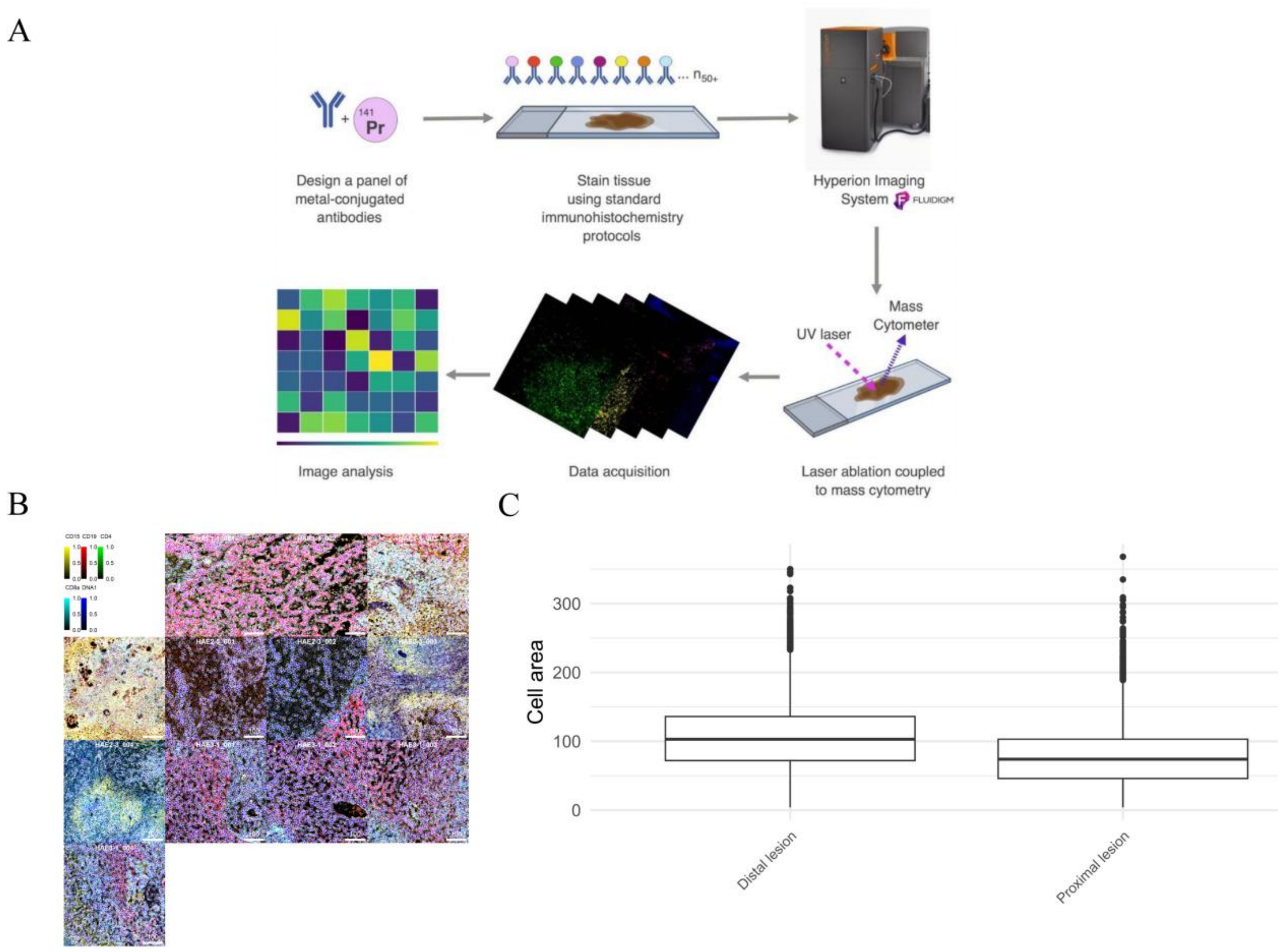

Supplementary Figure 1. Quality control of imaging mass cytometry (IMC) data. (A) Schematic diagram of the IMC experimental workflow, including metal-conjugated antibody panel design, tissue staining, Hyperion imaging system data acquisition, and image analysis. (B) Representative IMC pseudocolor images showing the expression patterns of multiple immune markers in HAE liver tissues. Scale bar: 100 μm. (C) Box plots comparing the cell area distribution between distal lesion and proximal lesion regions. No significant difference was observed in cell size between the two regions, indicating comparable data quality.

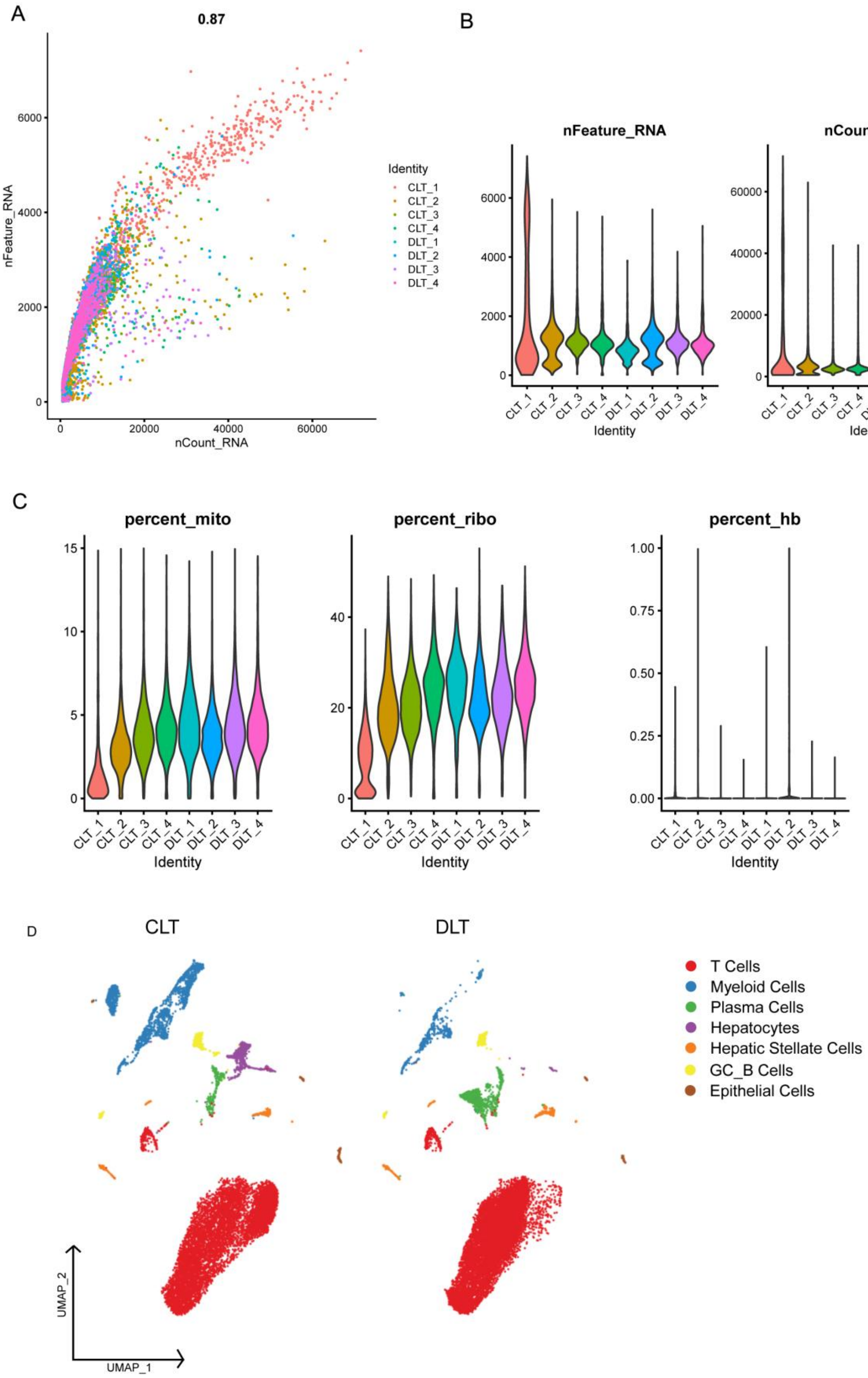

Supplementary Figure 2. Quality control and scRNA-seq integration. (A) Scatter plot showing the correlation between the number of detected genes (nFeature_RNA) and the number of unique molecular identifiers (nCount_RNA) per cell across all samples. The Pearson correlation coefficient is 0.87, indicating high overall data quality. (B) Violin plots showing the distribution of nFeature_RNA and nCount_RNA after quality control filtering, demonstrating comparable sequencing depth across

samples (CLT_1–4 and DLT_1–4). (C) Distribution of mitochondrial (percent_mito), ribosomal (percent_ribo), and hemoglobin (percent_hb) gene expression percentages across all samples, confirming effective filtering of low-quality cells and stressed hepatocytes. (D) Split UMAP visualization showing the distribution of major immune and stromal cell clusters in CLT and DLT regions, confirming balanced sample integration without significant batch effects.

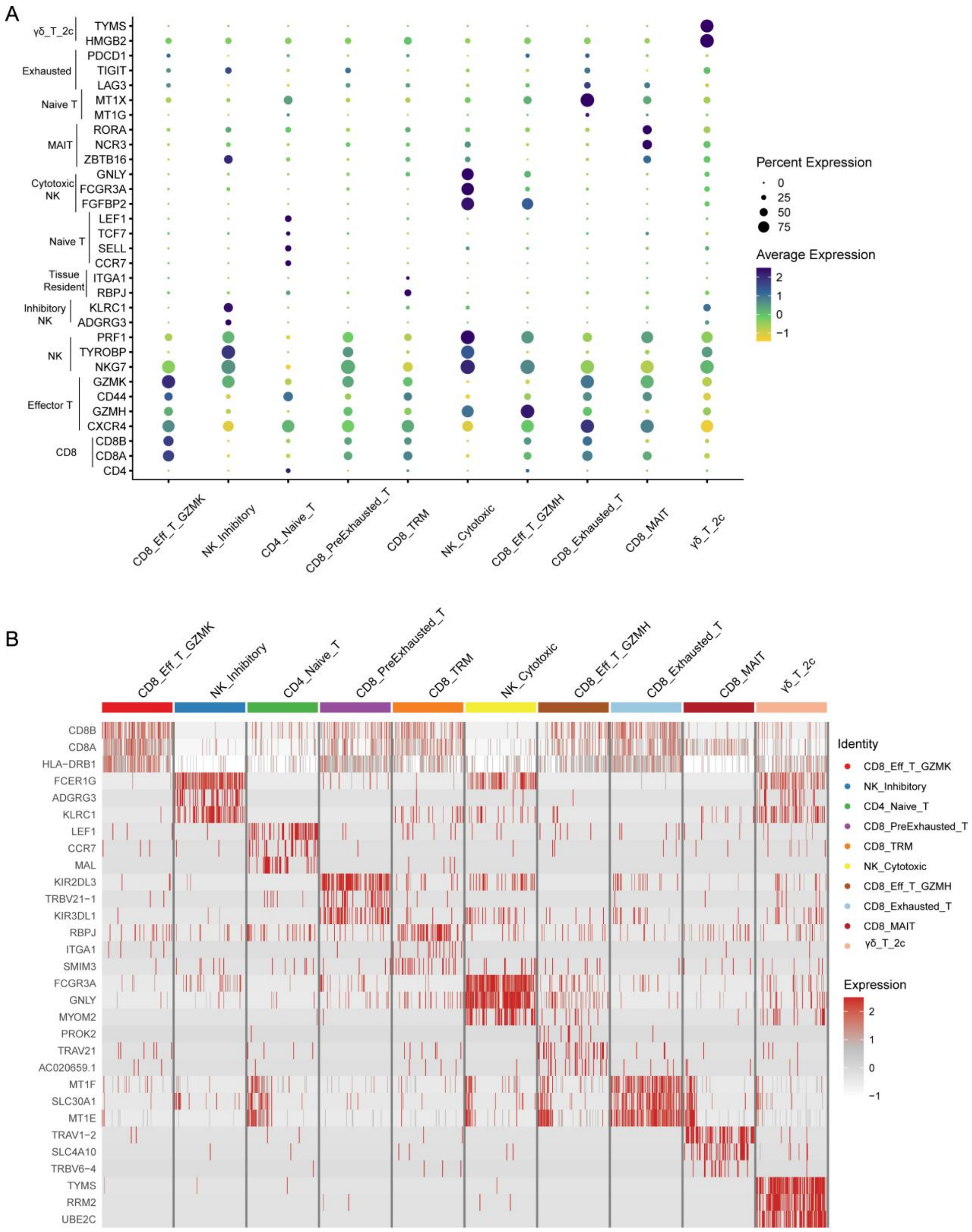

Supplementary Figure 3. Transcriptional profiling and marker gene identification of T and NK cell subsets. (A) Dot plot showing the expression of representative marker genes for each T and NK cell subset identified in Figure 2. The dot size represents the percentage of cells expressing the gene, and the color intensity represents the average expression level (scaled z-score). (B) Heatmap illustrating the top differentially expressed genes (DEGs) across all T and NK cell subsets. Each column represents an individual cell, and rows represent selected marker genes. The color scale indicates the normalized expression levels (z-score). Clusters are color-coded as shown in the identity legend.

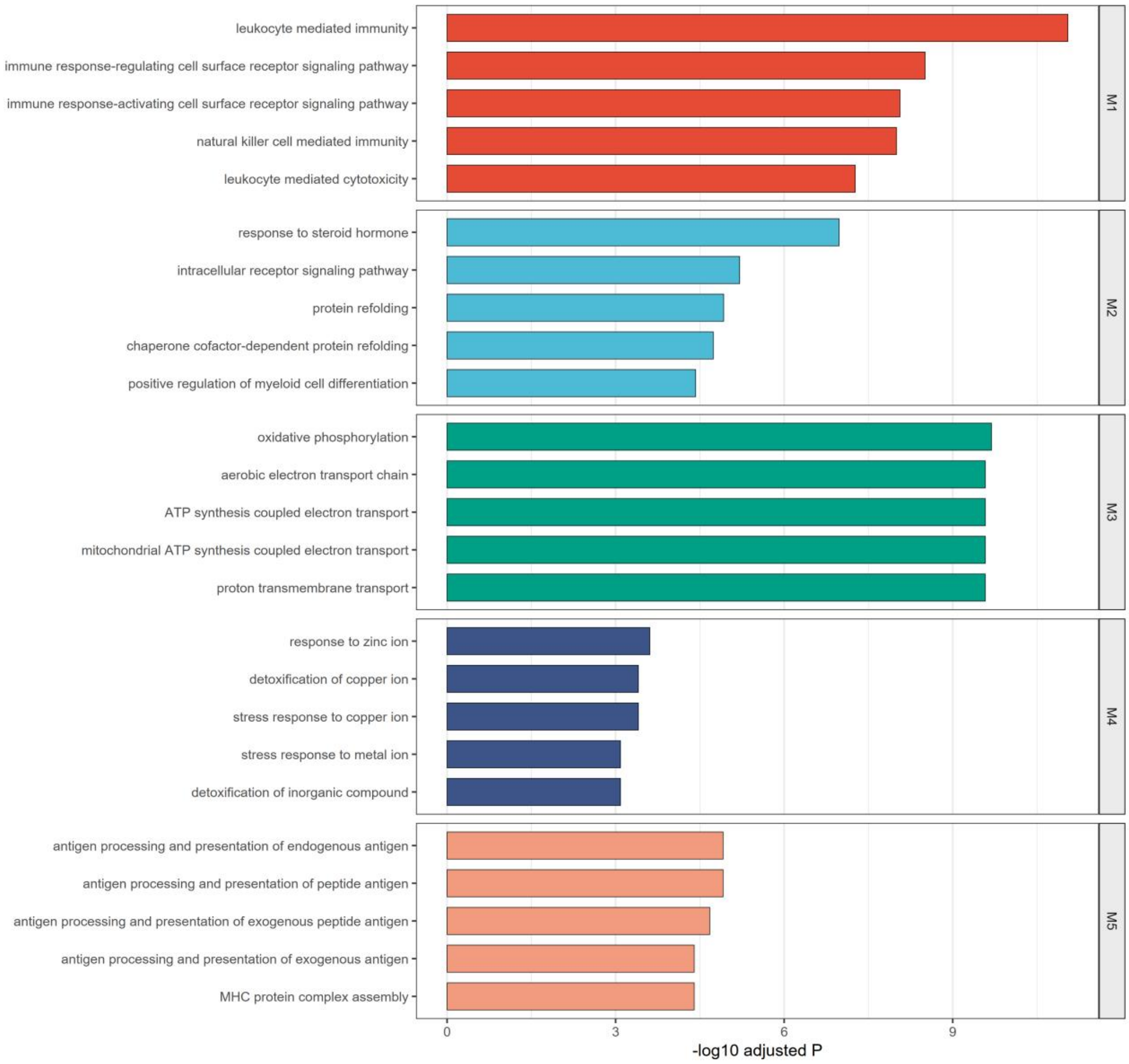

Supplementary Figure 4. Functional enrichment analysis of gene clusters identified in the T and NK cell trajectory. Gene Ontology (GO) biological process enrichment analysis of the five gene expression clusters (M1–M5, corresponding to clusters C1–C5 in Figure 3D) identified along the pseudotime trajectory of T/NK cell differentiation. The horizontal axis represents the significance level, expressed as −log10 (adjusted P-value). The top five enriched biological processes for each cluster are shown. M1 is primarily associated with immune effector functions and cytotoxicity; M2 with protein folding and signaling regulation; M3 with mitochondrial oxidative phosphorylation and energy metabolism; M4 with metal ion detoxification and stress responses; and M5 with antigen processing and MHC-mediated presentation.

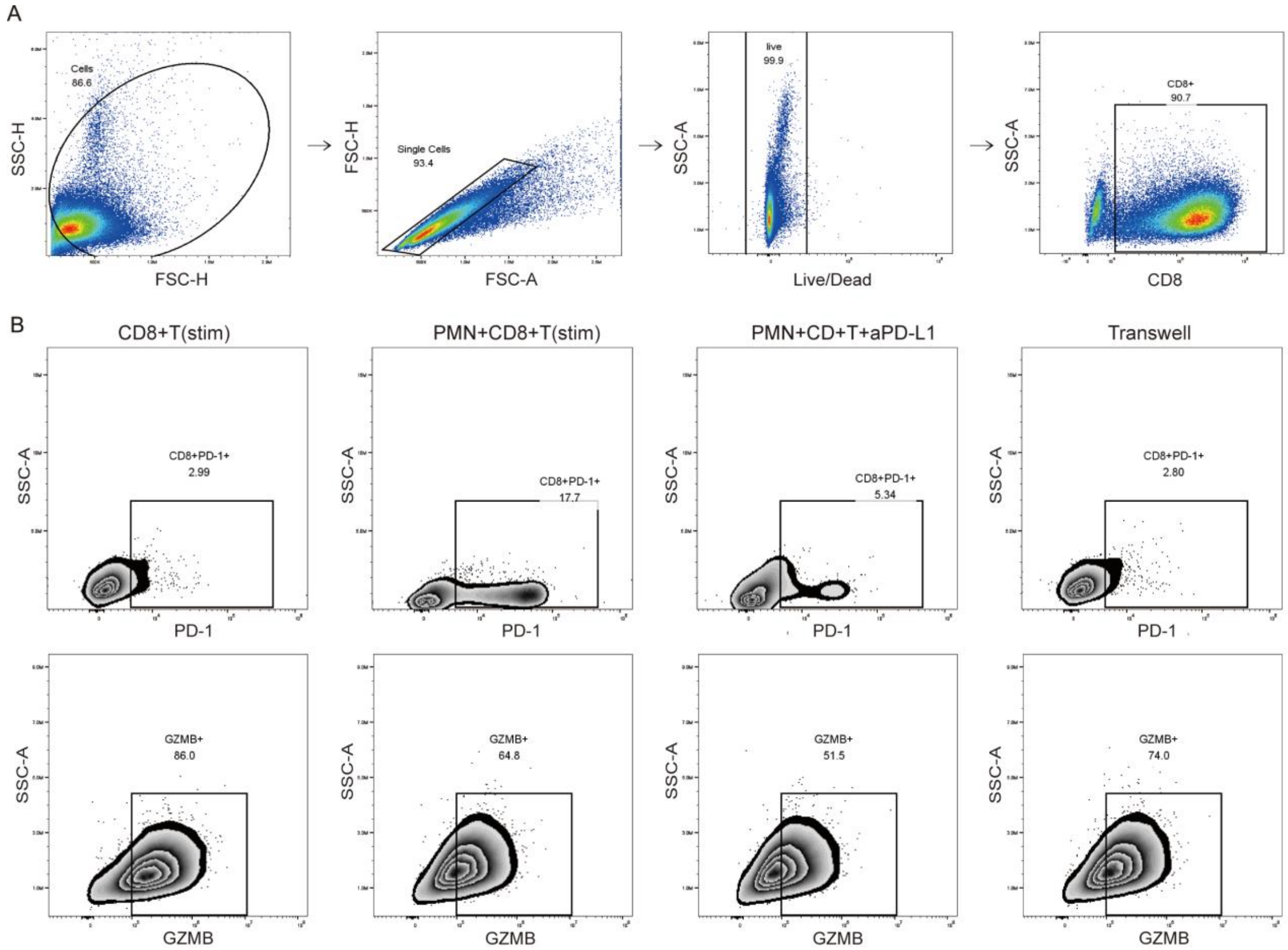

Supplementary Figure 5. Flow cytometry gating strategy and representative plots for the in vitro co-culture model. (A) Representative flow cytometry gating strategy used to isolate $CD8^+$ T cells. The selection criteria included sequential gating on cells (SSC-H vs. FSC-H), single cells (FSC-H vs. FSC-A), live cells (Live/Dead staining), and $CD8^+$ T cells (CD8 expression). (B) Representative flow cytometry plots illustrating the frequencies of PD-1^+ and $GZMB^+$ $CD8^+$ T cells across the experimental groups: $CD8^+$ T (stim), PMN + $CD8^+$ T (stim), PMN + $CD8^+$ T + anti-PD-L1, and Transwell co-culture. These plots demonstrate the PMN-induced upregulation of the exhaustion marker PD-1 and the concurrent downregulation of the effector molecule GZMB, as well as the partial rescue effect achieved by PD-L1 blockade or physical separation (Transwell). Quantitative data are presented in Figure 6.

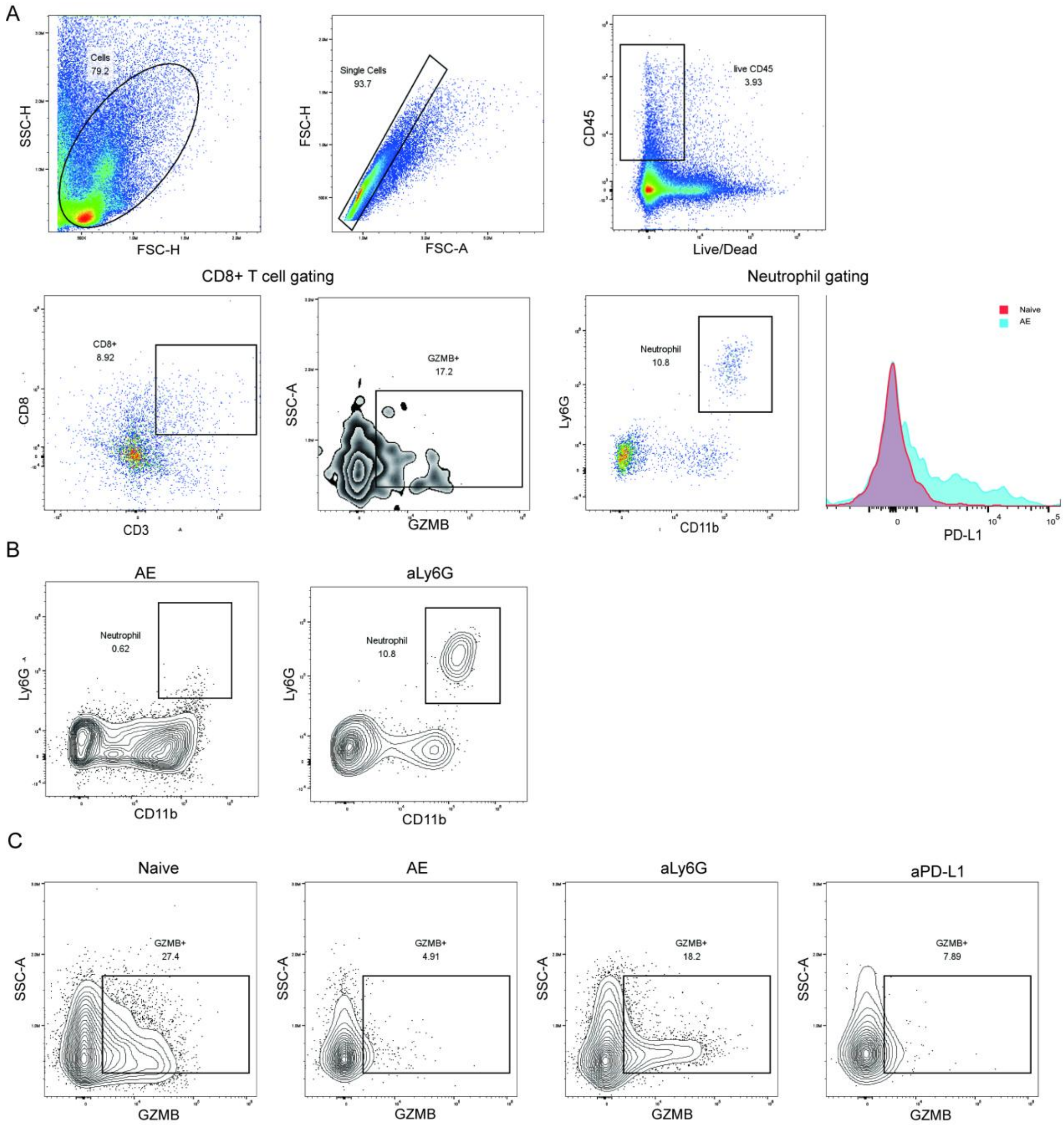

Supplementary Figure 6. Flow cytometry gating strategy and validation of in vivo interventions in HAE mouse model. (A) Representative gating strategy for hepatic immune cells. Cells were sequentially gated on cells (SSC-H vs. FSC-H), single cells (FSC-H vs. FSC-A), and live $CD45^+$ leukocytes, followed by identification of $CD8^+$ T cells ($CD3^+CD8^+$) and neutrophils ($CD11b^+Ly6G^+$). The histogram on the right compares PD-L1 expression levels on neutrophils between Naive and AE-infected mice. (B) Validation of neutrophil depletion efficiency. Representative flow plots showing the reduction of hepatic neutrophils ($CD11b^+Ly6G^+$) in the α-Ly6G-treated group compared with the untreated AE group, confirming the efficacy of the depletion strategy. (C) Representative flow cytometry plots assessing the frequency of $GZMB^+$ $CD8^+$ T cells across experimental groups (Naive, AE, α-Ly6G, and α-PD-L1). The data demonstrate the restoration of $CD8^+$ T cell cytotoxic function (indicated by GZMB expression) following neutrophil depletion or PD-L1 blockade. Quantitative data are presented in Figure 7.

Supplementary Table S1. Individual Clinical Data and Sample Usage of All Enrolled HAE Patients

Patient ID	Age (y)	Sex	PNM Stage	Lesion Max Diameter (cm)	Prior Albendazole Treatment	Disease Duration (months)	ALT (U/L)	AST (U/L)	Albumin (g/L)	Experiments Used
736518	33	F	P4N1M0	16.0	Yes (84 months)	84	32	31	37.6	IMC
738373	25	M	P4N1M1	17.0	No	8	151	159	39.4	IMC
735914	45	F	P3N1M0	13.0	No	12	37	27	39.7	IMC
611251	43	M	P4N0M0	15.0	No	36	46	35	29.2	scRNA-seq
611367	29	F	P2N0M0	20.0	Yes (120 months)	120	38	19	34.2	scRNA-seq
621884	58	M	P2N0M0	13.0	Yes (72 months)	72	34	20	36.6	scRNA-seq
617757	53	F	P2N0M0	12.0	Yes (84 months)	84	34	26	35.4	scRNA-seq
812099	22	F	P2N0M0	16.5	No	36	18	20	39.0	mIF/HE:
812437	9	M	P4N0M0	16.0	No	36	39	33	41.5	mIF/HE:
883927	50	M	P2N0M0	12.0	No	12	71	92	30.3	mIF/HE:
807204	27	M	P2N0M0	25.0	No	1	49	43	28.8	mIF/HE:
828947	48	F	P3N0M0	16.0	Yes (180 months)	180	20	33	26.9	mIF/HE:
**Summary**	**36.8 ± 15.2**	**6M/6F**	**-**	**15.8 ± 3.8**	**5/12 (41.7%)**	**36.0 (12.0-84.0)**	**37.5 (33.0-47.5)**	**32.0 (23.0-39.0)**	**34.8 ± 4.5**	**IMC: n=3; scRNA-seq: n=4; mIF/HE: n=5**

Note: All patients provided paired invasive margin tissue (CLT) and distal normal liver tissue (DLT) samples. Abbreviations: IMC, Imaging Mass Cytometry; scRNA-seq, single-cell RNA sequencing; mIF, multiplex immunofluorescence; HE, hematoxylin-eosin. "-" indicates the sample was not used in any of the listed experiments. All statistical summaries were calculated based on the 12 enrolled patients.

Supplementary Table S2. Top 11 Differentially Expressed Immune Markers Between CLT and DLT by Imaging Mass Cytometry

Marker	CLT (log_2, mean ± SD)	DLT (log_2, mean ± SD)	Cohen's d (95% CI)	Raw P	FDR
PD-1)	1.12 ± 0.61	-0.66 ± 0.12	1.92 (0.54, 3.21)	0.006	0.216
CD19	0.43 ± 0.09	0.78 ± 0.15	-1.80 (-3.07, -0.46)	0.009	0.216
IL-17A	0.51 ± 0.15	-0.18 ± 0.08	1.76 (0.42, 3.03)	0.010	0.216
CD15	0.49 ± 0.28	-0.84 ± 0.10	1.70 (0.38, 2.96)	0.012	0.216
PD-L2	0.13 ± 0.05	-0.39 ± 0.06	1.56 (0.25, 2.81)	0.019	0.216
CD56	0.10 ± 0.08	-1.30 ± 0.09	1.44 (0.14, 2.69)	0.028	0.216
CD45RO	0.11 ± 0.03	-0.75 ± 0.10	1.40 (0.10, 2.63)	0.032	0.216
CD11c	0.09 ± 0.02	-0.27 ± 0.04	1.36 (0.06, 2.58)	0.037	0.216
Bcl-6	0.10 ± 0.03	-0.58 ± 0.04	1.32 (0.03, 2.54)	0.041	0.216
CD8a	0.32 ± 0.21	-1.17 ± 0.15	1.29 (0.00, 2.50)	0.046	0.216
PD-L1	0.09 ± 0.04	-0.29 ± 0.07	1.27 (0.01, 2.49)	0.048	0.216

Notes: Only markers with unadjusted $P < 0.05$ are shown. Complete statistical results for all 28 immune markers are provided in Supplementary Data S1, Sheet 2: IMC Data.

Expression values are presented as $log_2(x+1)$ transformed intensity. Descriptive statistics (mean ± SD) are calculated based on the average of two ROIs per patient (patient-level, n=3 patients). Inferential statistics were generated using a linear mixed-effects model on 6 paired ROIs, with tissue region as fixed effect and patient as random intercept. FDR correction was performed using the Benjamini-Hochberg method across all 28 markers.

Supplementary Table S3. Antibody Panel for Imaging Mass Cytometry

Category	Marker	Specificity	Clone	Metal Tag	Supplier
**Pan-immune Marker**	PTPRC (CD45)	Pan-leukocytes	D9M8I	Dy163	Cell Signaling Technology
**T Lymphocytes**	CD3E (CD3)	T and NKT cells	Polyclonal (C-Terminal)	Er170	Cell Signaling Technology
	CD4	$CD4^{+}$ T cells	EPR6855	Gd156	Abcam
	CD8A (CD8a)	$CD8^{+}$ T cells	C8/144B	Dy162	Dako
	PTPRCRA (CD45RA)	Naïve T cells	MEM-56	Nd143	Thermo Fisher
	PTPRCRO (CD45RO)	Memory T cells	UCH-L1	Er166	Dako
	FOXP3	Regulatory T cells (Tregs)	206D	Gd155	BioLegend
	TBX21 (T-bet)	Th1 lineage transcription factor	4B10	Lu175	Cell Signaling Technology
	GATA3	Th2 lineage transcription factor	EPR16651	Yb176	Abcam
	BCL6	Tfh lineage transcription factor	KA4B18	Er167	BioLegend
**B Lymphocytes**	CD19	B cells	6OMP31	Nd142	Cell Signaling Technology
**Myeloid Cells**	CD11C (ITGAX)	Dendritic cells	EP1347Y	Sm154	Abcam
	CD68	Macrophages	KP1	Tb159	Dako
	CD15 (SSEA1)	Granulocytes/Neutrophils	HI98	Eu153	BioLegend
	NCAM1 (CD56)	NK and NKT cells	RNL-1	Sm152	Thermo Fisher
	CD33	Myeloid progenitor cells	WM53	Nd145	BD Biosciences
**Fibrosis & Stroma**	ACTA2 (α-SMA)	Myofibroblasts/Hepatic stellate cells	1A4	Pr141	Sigma-Aldrich
	COL1A1 (Collagen I)	Type I collagen	COL-1	Tm169	Abcam
	TGFB1 (TGF-β)	Profibrotic cytokine	TW4-2H10	Eu151	R&D Systems

Category	Marker	Specificity	Clone	Metal Tag	Supplier
**Proliferation & Activation**	MKI67 (Ki-67)	Cellular proliferation marker	B56	Er168	BD Biosciences
	HLA-DRA (HLA-DR)	Antigen presentation	YE2/36 HLK	Yb174	BioLegend
**Effector Cytokines**	IFNG (IFN-γ)	Th1 effector cytokine	4S.B3	Sm147	BioLegend
	TNF (TNF-α)	Proinflammatory cytokine	MAb11	Gd160	BioLegend
	IL6	Proinflammatory cytokine	MQ2-13A5	Nd148	BioLegend
	IL17A (IL-17a)	Th17 effector cytokine	BL168	Gd158	BioLegend
**Immune Checkpoints**	CD274 (PD-L1)	Programmed death-ligand 1	E1L3N	Nd150	Cell Signaling Technology
	PDCD1LG2 (PD-L2)	Programmed death-ligand 2	176611	Yb172	R&D Systems
	PDCD1 (PD-1)	Programmed cell death protein 1	EPR4877(2)	Ho165	Abcam

Note: This panel includes a total of 28 antibodies targeting key immune cell subsets, functional molecules and stromal markers. All antibodies were validated for immunohistochemistry and imaging mass cytometry according to the manufacturer's instructions. Metal tags were conjugated using the Maxpar X8 Antibody Labeling Kit (Fluidigm).

Supplementary Table S4. Differentially Expressed Markers (PreExhausted vs GZMK^{+} CD8^{+} T cells)

Rank	Gene Symbol	*avg_log₂FC*	Raw *P* Value	Adjusted *P* Value	pct.1 (PreExhausted)	pct.2 (GZMK^{+})	Gene Function	Cell Specificity
1	KIR2DL1	3.87	1.21×10^{-98}	2.43×10^{-94}	0.89	0.07	Killer cell immunoglobulin-like receptor; inhibits T cell activity	PreExhausted-enriched
2	PDCD1	3.55	4.56×10^{-92}	9.12×10^{-88}	0.85	0.11	Programmed cell death 1; core marker of T cell exhaustion	PreExhausted-enriched
3	TOX	3.22	7.89×10^{-85}	1.58×10^{-81}	0.82	0.13	Transcription factor; drives T cell exhaustion program	PreExhausted-enriched
4	LAG3	2.99	1.34×10^{-79}	2.68×10^{-75}	0.78	0.15	Immune checkpoint; enhances T cell suppression	PreExhausted-enriched
5	HAVCR2	2.76	5.67×10^{-73}	1.13×10^{-69}	0.75	0.18	TIM-3 receptor; mediates T cell exhaustion	PreExhausted-enriched
6	GZMK	-3.98	2.34×10^{-102}	4.68×10^{-98}	0.09	0.92	Granzyme K; cytotoxic marker of effector T cells	GZMK^{+}-enriched
7	PRF1	-3.65	6.78×10^{-95}	1.36×10^{-91}	0.12	0.88	Perforin; mediates cytotoxic killing	GZMK^{+}-enriched
8	NKG7	-3.25	9.01×10^{-88}	1.80×10^{-84}	0.15	0.85	NK granule protein; enhances effector function	GZMK^{+}-enriched
9	CCL4	-2.98	3.45×10^{-82}	6.90×10^{-78}	0.18	0.81	Chemokine; recruits immune cells	GZMK^{+}-enriched
10	CX3CR	-2.76	$5.67\times10^{-}$	1.13×10	0.21	0.78	Chemokine	GZMK^{+}-enriched

Rank	Gene Symbol	*avg_log_2FC*	Raw *P* Value	Adjusted *P* Value	pct.1 (PreExhausted)	pct.2 ($GZMK^+$)	Gene Function	Cell Specificity
	1		76	−72			receptor; regulates T cell migration	
11	TIGIT	2.54	8.90×10^{-70}	1.78×10^{-66}	0.72	0.23	Immune checkpoint; inhibits T cell activation	PreExhausted-enriched
12	CTLA4	2.32	1.23×10^{-65}	2.46×10^{-61}	0.68	0.25	Immune checkpoint; downregulates T cell function	PreExhausted-enriched
13	IFNG	-2.11	4.56×10^{-60}	9.12×10^{-56}	0.28	0.75	Interferon-γ; enhances effector immunity	$GZMK^+$-enriched
14	TNF	-1.99	7.89×10^{-55}	1.58×10^{-51}	0.31	0.72	Tumor necrosis factor; mediates inflammation	$GZMK^+$-enriched
15	IL2RA	-1.77	1.34×10^{-50}	2.68×10^{-46}	0.35	0.68	IL-2 receptor α; regulates T cell proliferation	$GZMK^+$-enriched
16	CD28	-1.54	5.67×10^{-45}	1.13×10^{-41}	0.38	0.65	T cell co-stimulation; enhances activation	$GZMK^+$-enriched
17	CD3E	0.90	9.01×10^{-40}	1.80×10^{-36}	0.95	0.93	TCR component; pan-T cell marker	Co-expressed
18	CD8A	0.77	3.45×10^{-35}	6.90×10^{-31}	0.94	0.92	CD8 α chain; $CD8^+$ T cell marker	Co-expressed
19	CD3D	0.65	5.67×10^{-30}	1.13×10^{-26}	0.96	0.94	TCR component; pan-T cell marker	Co-expressed
20	CD2	0.55	8.90×10^{-25}	1.78×10^{-21}	0.93	0.91	T cell adhesion molecule	Co-expressed

Note: Differential gene expression analysis was performed using the Wilcoxon rank-sum test in Seurat v4.3.0. Genes were filtered using the criteria of adjusted P value < 0.05 and |*avg_log_2FC*| > 0.5. The top 20 genes were ranked in descending order

of |avg_log_2FC|. pct.1 and pct.2 indicate the percentage of cells expressing the gene in PreExhausted and $GZMK^+$ $CD8^+$ T cells, respectively. Positive avg_log_2FC values indicate higher expression in PreExhausted cells; negative values indicate higher expression in $GZMK^+$ cells. A total of 327 differentially expressed genes were identified. Functional enrichment analysis of these genes is provided in Supplementary Table S5.

Supplementary Table S5. Functional Enrichment Analysis of Genes During GZMK⁺ to PreExhausted CD8⁺ T Cell Transition

S5-1. Gene Ontology Biological Process (Top 15, adjusted P < 0.05)

Rank	Pathway ID	Pathway Name	Gene Count	Background Genes	Fold Enrichment	Raw *P* Value	Adjusted *P* Value	Core Genes
1	GO:0002262	Negative regulation of T cell activation	48	326	8.92	1.21×10^{-38}	2.43×10^{-34}	PDCD1, LAG3, TIGIT, CTLA4
2	GO:0002821	Immune suppression	42	289	8.56	4.56×10^{-35}	9.12×10^{-31}	TOX, HAVCR2, KIR2DL1
3	GO:0002446	Negative regulation of immune response	56	412	7.98	7.89×10^{-32}	1.58×10^{-28}	PDCD1, LAG3, TGFBR1
4	GO:0030098	Lymphocyte differentiation	63	487	7.65	1.34×10^{-29}	2.68×10^{-25}	TOX, CD8A, IL2RA
5	GO:0042110	T cell activation	72	593	7.23	5.67×10^{-27}	1.13×10^{-23}	CD28, ZAP70
6	GO:0006955	Immune response	89	756	6.89	9.01×10^{-25}	1.80×10^{-21}	IFNG, TNF, IL2
7	GO:0002376	Immune system process	95	821	6.54	3.45×10^{-22}	6.90×10^{-18}	CD8A, CD4, CD3E
8	GO:0001916	T cell-mediated cytotoxicity	38	315	6.21	5.67×10^{-20}	1.13×10^{-16}	GZMK, PRF1, IFNG
9	GO:0002520	Immune effector process	67	568	5.98	8.90×10^{-18}	1.78×10^{-14}	TNF, IL2, IFNG
10	GO:0045087	Innate immune response	52	443	5.67	1.23×10^{-15}	2.46×10^{-11}	NKG7, CX3CR1
11	GO:0002437	APC-mediated T cell activation	45	389	5.34	4.56×10^{-13}	9.12×10^{-09}	CD28, CTLA4
12	GO:0019882	Antigen processing and presentation	39	336	5.01	7.89×10^{-11}	1.58×10^{-07}	HLA-DRA, TAP1

Rank	Pathway ID	Pathway Name	Gene Count	Background Genes	Fold Enrichment	Raw *P* Value	Adjusted *P* Value	Core Genes
13	GO:0002250	Adaptive immune response	58	502	4.89	1.34×10^{-09}	2.68×10^{-05}	CD8A, PDCD1
14	GO:0002819	Regulation of immune response	47	405	4.67	5.67×10^{-08}	1.13×10^{-04}	TGFBR1, CTLA4
15	GO:0006950	Stress response	76	689	4.32	9.01×10^{-06}	1.80×10^{-02}	HSPA1A, TNF

S5-2 Reactome Pathway (Top 10, adjusted p < 0.05)

Rank	Pathway ID	Pathway Name	Gene Count	Background Genes	Fold Enrichment	Raw P Value	Adjusted P Value	Core Genes
1	R-HSA-166658	PD-L1/PD-1 signaling	28	196	9.23	1.21×10^{-35}	2.43×10^{-31}	PDCD1, CD274, STAT3
2	R-HSA-202403	T cell receptor signaling	36	258	8.76	4.56×10^{-32}	9.12×10^{-28}	CD3D, LCK, ZAP70
3	R-HSA-1280218	Immune checkpoint inhibition	24	172	8.34	7.89×10^{-29}	1.58×10^{-25}	PDCD1, LAG3, TIGIT
4	R-HSA-198933	Interleukin signaling	32	231	7.98	1.34×10^{-26}	2.68×10^{-22}	IL2RA, IL6R
5	R-HSA-168256	Chemokine signaling	29	207	7.56	5.67×10^{-23}	1.13×10^{-19}	CX3CR1, CCL4
6	R-HSA-116909	NF-κB signaling	34	245	7.21	9.01×10^{-21}	1.80×10^{-17}	TNF, RELA
7	R-HSA-1266690	Interferon signaling	27	192	6.89	3.45×10^{-18}	6.90×10^{-14}	IFNG, STAT1
8	R-HSA-2162773	Cytokine-receptor interaction	41	302	6.54	5.67×10^{-16}	1.13×10^{-12}	TNF, IL2
9	R-HSA-109582	Complement system	18	136	6.21	8.90×10^{-13}	1.78×10^{-09}	C3, C5
10	R-HSA-164843	Phosphoinositide signaling	25	185	5.98	1.23×10^{-10}	2.46×10^{-06}	PLCG1, AKT1

Note: Functional enrichment analysis was performed using clusterProfiler v4.6.2. The background gene set was all detected genes in the $CD8^+$ T cell subset. Multiple comparisons were corrected using the Benjamini-Hochberg method. Core genes were selected based on the magnitude of differential expression and biological relevance. Differentially expressed genes used for enrichment analysis are provided in Supplementary Table S4.

Supplementary Table S6. Cell Subset Annotation for Single-Cell RNA Sequencing

Cluster ID	Cell Type Annotation	Top 5 Marker Genes	Annotation Basis
0	PreExhausted CD8⁺ T cells	PDCD1, TOX, LAG3, HAVCR2, TIGIT	Canonical exhaustion markers + manual curation
1	GZMK⁺ effector CD8⁺ T cells	GZMK, PRF1, NKG7, CCL4, CX3CR1	Canonical effector T cell markers + manual curation
2	PD-L1⁺ neutrophils	CD15, S100A8, S100A9, CD274, CSF3R	Granulocyte markers + PD-L1 expression
3	CD4⁺ T cells	CD4, IL7R, TRAC, CD3D, CD3E	Canonical CD4⁺ T cell markers
4	Regulatory T cells (Tregs)	FOXP3, IL2RA, CTLA4, TNFRSF18, IKZF2	Canonical Treg markers
5	B cells	CD19, MS4A1, CD79A, CD79B, IGHD	Canonical B cell markers
6	Monocytes/macrophages	CD68, CD14, CSF1R, APOE, C1QA	Canonical myeloid markers
7	Dendritic cells	CD1C, CLEC9A, HLA-DRA, CD83, FCER1A	Canonical dendritic cell markers
8	NK cells	NCAM1, KLRD1, GNLY, NKG7, FCGR3A	Canonical NK cell markers
9	Hepatic stellate cells	ACTA2, COL1A1, COL3A1, TGFB1, DES	Canonical stromal/fibroblast markers

Note: Single-cell RNA sequencing data were processed and analyzed using Seurat v4.3.0. Cell clustering was performed using the Louvain algorithm with a resolution of 0.5. Marker genes for each cluster were identified using the Wilcoxon rank-sum test with the criteria of adjusted P value < 0.05 and |*avg_log_2FC*| > 0.5. All annotations were manually verified based on published literature and canonical immune cell markers. Differential gene expression analysis between PreExhausted and GZMK⁺ CD8⁺ T cells is provided in Supplementary Table S4.

Supplementary Table S7. Liver pathology and immune parameters in AE mice treated with anti-Ly6G or anti-PD-L1

Parameter	Naive (n=6/3*)	AE (n=6/3*)	α- Ly6G (n=6/9*)	α- PD- L1 (n=6/3*)
Lesion area (%)	0.00±0.00	46.65±0.79	20.27±0.65**	39.66±0.88*
Inflammation score (0-4)	0.00±0.00	3.83±0.11	1.33±0.17**	2.67±0.17**
Fibrosis score (0-4)	0.00±0.00	3.83±0.21	1.17±0.11**	2.58±0.15**
$CD8^+/CD45^+$ (%)	36.13±6.23	30.91±2.52	8.62±3.87**	27.71±2.58
$CD8^+GZMB^+$ (%)	10.00±3.67	3.03±0.81	26.33±6.55*	18.65±5.62*
$CD8^+IFN\text{-}\gamma^+$ (%)	20.17±0.33	15.27±0.87	12.71±2.09	16.10±0.87
$CD8^+TOX^+$ (%)	8.00±0.73	27.40±2.50**	23.32±7.37	20.37±1.62*
$CD8^+Tex^+$ (%)	4.09±0.43	11.98±1.76**	12.48±8.06	6.01±1.84*
Neutrophil (%)	5.24±1.27	6.34±2.22	0.80±0.11**	5.47±0.23
Neutrophil PD- $L1^+$ (%)	9.31±1.00	34.90±3.12**	7.57±1.27**	19.17±2.61*

All data are presented as mean ± standard error of the mean (SEM).

*Sample size notation: n=6 for lesion area, inflammation score and fibrosis score (6 mice per group); n=3 for flow cytometry analysis (3 mice per group), except for the anti-Ly6G group (n=9, pooled from two independent experiments).

Statistical analysis was performed using one-way ANOVA followed by Tukey's HSD post-hoc test for comparisons across four groups.

*$P < 0.05$, **$P < 0.01$ compared with the untreated AE group.

Abbreviations: AE, hepatic alveolar echinococcosis; GZMB, granzyme B; IFN-γ, interferon-gamma; TOX, thymocyte selection-associated high mobility group box protein; Tex, exhausted $CD8^+$ T cells.

Supplementary Table S8: qPCR Primer Sequences and Reaction Conditions

Part 1: Primer Sequences

Gene Symbol	Primer Direction	Sequence (5' → 3')	Amplicon Length (bp)	Annealing Temperature (°C)
GAPDH	Forward (F)	GGTCGGAGTCAACGGATTTG	185	60
GAPDH	Reverse (R)	GGAAGATGGTGATGGGATTTC	185	60
TOX	Forward (F)	GAGGCTGCGAAGAAGGAGTA	162	60
TOX	Reverse (R)	GCTGAGGAGGTTGAGATGTCT	162	60
NR4A1	Forward (F)	GTACATCTGCCTGGCTAACAAG	197	60
NR4A1	Reverse (R)	TCGGACAACTTCCTTCACCAT	197	60
GZMB	Forward (F)	GTGCGGTGGCTTCCTGATA	173	60
GZMB	Reverse (R)	GGGTCGGCTCCTGTTCTTT	173	60
PRF1	Forward (F)	GCCTGTGAGGAGAAGAAGAAGA	204	60
PRF1	Reverse (R)	GTCGTTAATGGAGGTGTGATGG	204	60

Note: GAPDH was used as the endogenous reference gene for normalization.

Part 2: qPCR Reaction Conditions

Program Step	Temperature	Time	Number of Cycles
Initial Denaturation	95°C	5 min	1
Denaturation	95°C	15 sec	40
Annealing & Extension	60°C	30 sec	40
Melting Curve Analysis	95°C	15 sec	1
	60°C	60 sec	1
	95°C	15 sec	1

Note: All reactions were performed using a standard SYBR Green-based qPCR protocol. Relative gene expression was calculated using the 2^(-ΔΔCt) method.For quantitative real-time PCR analysis of immune-related gene expression in co-cultured cells
