# Supplementary material for: PD-L1+ neutrophils at the invasive margin drive contact-dependent CD8+ T cell exhaustion in hepatic alveolar echinococcosis
Source: Front Immunol. 2026 Sep 10;17:1917606. doi: 10.3389/fimmu.2026.1917606 (PMC13600833; doi:10.3389/fimmu.2026.1917606)
Supplement: Supplementary file 2 [file Table2.docx]

**Supplementary Table S3-1. Top 11 Differentially Expressed Immune Markers Between CLT and DLT by Imaging Mass Cytometry**

| **Marker** | **CLT**  **(log₂, mean ± SD)** | **DLT**  **(log₂, mean ± SD)** | **Cohen's d**  **(95% CI)** | **Raw P** | **FDR** |
| --- | --- | --- | --- | --- | --- |
| PD-1) | 1.12 ± 0.61 | -0.66 ± 0.12 | 1.92 (0.54, 3.21) | 0.006 | 0.216 |
| CD19 | 0.43 ± 0.09 | 0.78 ± 0.15 | -1.80 (-3.07, -0.46) | 0.009 | 0.216 |
| IL-17A | 0.51 ± 0.15 | -0.18 ± 0.08 | 1.76 (0.42, 3.03) | 0.010 | 0.216 |
| CD15 | 0.49 ± 0.28 | -0.84 ± 0.10 | 1.70 (0.38, 2.96) | 0.012 | 0.216 |
| PD-L2 | 0.13 ± 0.05 | -0.39 ± 0.06 | 1.56 (0.25, 2.81) | 0.019 | 0.216 |
| CD56 | 0.10 ± 0.08 | -1.30 ± 0.09 | 1.44 (0.14, 2.69) | 0.028 | 0.216 |
| CD45RO | 0.11 ± 0.03 | -0.75 ± 0.10 | 1.40 (0.10, 2.63) | 0.032 | 0.216 |
| CD11c | 0.09 ± 0.02 | -0.27 ± 0.04 | 1.36 (0.06, 2.58) | 0.037 | 0.216 |
| Bcl-6 | 0.10 ± 0.03 | -0.58 ± 0.04 | 1.32 (0.03, 2.54) | 0.041 | 0.216 |
| CD8a | 0.32 ± 0.21 | -1.17 ± 0.15 | 1.29 (0.00, 2.50) | 0.046 | 0.216 |
| PD-L1 | 0.09 ± 0.04 | -0.29 ± 0.07 | 1.27 (0.01, 2.49) | 0.048 | 0.216 |

Notes: Only markers with unadjusted P < 0.05 are shown. Complete statistical results for all 28 immune markers are provided in Supplementary Data S1, Sheet 2: IMC Data.

Expression values are presented as log₂(x+1) transformed intensity. Descriptive statistics (mean ± SD) are calculated based on the average of two ROIs per patient (patient-level, n=3 patients). Inferential statistics were generated using a linear mixed-effects model on 6 paired ROIs, with tissue region as fixed effect and patient as random intercept. FDR correction was performed using the Benjamini-Hochberg method across all 28 markers.

**Supplementary Table S3-2. Spearman’s rank correlation coefficients (ρ) and corresponding P values for pairwise immune marker expression in collagenous lesion tissue (CLT) and distal normal liver tissue (DLT), n=6 regions of interest (ROIs) per tissue group.**

| **Marker Pair** | **CLT tissue – Spearman ρ** | **CLT P value** | **CLT Significance** | **DLT tissue – Spearman ρ** | **DLT P value** | **DLT Significance** |
| --- | --- | --- | --- | --- | --- | --- |
| PD-1 vs PD-L1 | 0.89 | 0.019 | * | 0.37 | 0.47 | ns |
| PD-1 vs PD-L2 | 0.83 | 0.042 | * | 0.31 | 0.55 | ns |
| PD-L1 vs PD-L2 | 0.94 | 0.005 | ** | — | — | — |
| PD-1 vs CD8a | 0.77 | 0.072 | Trend | — | — | — |
| CD8a vs T-bet | 0.89 | 0.019 | * | — | — | — |
| CD8a vs CD45RO | 0.83 | 0.042 | * | — | — | — |
| T-bet vs CD45RO | 0.94 | 0.005 | ** | — | — | — |
| CD11c vs CD15 | 0.77 | 0.072 | Trend | 0.89 | 0.019 | * |
| CD11c vs CD33 | — | — | — | 0.83 | 0.042 | * |
| CD19 vs CD68 | — | — | — | 0.77 | 0.072 | Trend |
| CD19 vs TGF-β | — | — | — | 0.71 | 0.11 | ns |
